# Ordered micro/macro porous K-OMS-2/SiO_2_ nanocatalysts: Facile synthesis, low cost and high catalytic activity for diesel soot combustion

**DOI:** 10.1038/srep43894

**Published:** 2017-04-26

**Authors:** Xuehua Yu, Zhen Zhao, Yuechang Wei, Jian Liu

**Affiliations:** 1 Institute of Catalysis for Energy and Environment, College of Chemistry and Chemical Engineering, Shenyang Normal University, Shenyang, Liaoning, 110034, China; 2 State Key Laboratory of Heavy Oil Processing, China University of Petroleum, 18# Fuxue Road, Chang Ping, Beijing 102249, China

## Abstract

A series of novel oxide catalysts, which contain three-dimensionally ordered macroporous (3DOM) and microporous structure, were firstly designed and successfully synthesized by simple method. In the as-prepared catalysts, 3DOM SiO_2_ is used as support and microporous K-OMS-2 oxide nanoparticles are supported on the wall of SiO_2_. 3DOM K-OMS-2/SiO_2_ oxide catalysts were firstly used in soot particle oxidation reaction and they show very high catalytic activities. The high activities of K-OMS-2/SiO_2_ oxide catalysts can be assigned to three possible reasons: macroporous effect of 3DOM structure for improving contact between soot and catalyst, microporous effect of K-OMS-2 for adsorption of small gas molecules and interaction of K and Mn for activation of gas molecules. The catalytic activities of catalysts are comparable to or even higher than noble metal catalyst in the medium and high temperature range. For example, the T_50_ of K-OMS-2/SiO_2_-50, 328 °C, is much lower than those of Pt/Al_2_O_3_ and 3DOM Au/LaFeO_3_, 464 and 356 °C,respectively. Moreover, catalysts exhibited high catalytic stability. It is attributed to that the K^+^ ions are introduced into the microporous structure of OMS-2 and stabilized in the catalytic reaction. Meanwhile, the K^+^ ions play an important role in templating and stabilizing the tunneled framework of OMS-2.

Soot particles derived from the exhaust of diesel engine are a kind of main source of urban atmospheric particulate matters, which is directly threatening environment and people’s health^1^,^2^. Therefore, elimination of soot particles is urgent for the protection of environment. In previous studies, many techniques have been developed for solving pollution of diesel engine exhaust, and the after-treatment of diesel exhaust is one of the most perspective techniques^3^,^4^. The process of soot combustion is a catalytic reaction involving three phases of gas(O_2_)-solid(soot)-solid(catalyst)^5^. It is affected by two factors: the contact condition between soot and catalyst, and the intrinsic activity of catalyst. Therefore, design and preparation of catalysts, which can effectively enhance contact area and intrinsic activity of catalysts, is significant for soot combustion.

Due to smaller pore sizes (<10 nm) of traditional catalysts than soot particles (>20 nm), it is difficult for soot particles to enter inner pores of these catalysts so that their catalytic activities are limited^6^. Thus, finding novel catalysts, which can effectively make use of inner surface for increasing the contact area between catalysts and soot particles, is a key factor affecting the catalytic activity for soot combustion. Recently, three-dimensionally ordered macroporous (3DOM) oxides have been considered as potential catalysts owing to uniform pore size (>50 nm) and well-defined structure^7^,^8^,^9^. The soot particles not only could enter their inner pores more easily, but also could access the active sites more flexibly than nanoparticle samples when 3DOM materials are taken as catalysts for soot combustion. In our previous studies, a series of 3DOM metal mixed oxides, including La_1-x_K_x_CoO_3_^10^ and Ce_1-x_Zr_x_O_2_^11^ and so on, have been prepared and they all show better catalytic performances than the corresponding nanoparticle catalysts for soot oxidation. However, because of limitation of intrinsic activity, 3DOM oxide-based catalysts do not show high enough activity for soot oxidation at low temperature. In order to further improve the intrinsic activity of catalysts for soot combustion, the advantages of supported noble metal (Au, Pt, Pd, Ag, etc) nanoparticles (NPs) catalysts have been recognized^12^,^13^. We have synthesized several kinds of 3DOM oxides-supported Au and Pt catalysts^14^. Those 3DOM oxides-supported noble metal catalysts exhibit high catalytic activities for soot combustion. However, noble metal catalysts are very expensive than metal oxide catalysts, which restricts the extensive employment of noble metal catalysts.

Novel catalysts with low cost and fine intrinsic activity are significant for practical application^15^,^16^. Due to three phases of gas (O_2_)-solid (soot)-solid (catalyst) for catalytic soot combustion, adsorption and activation of small gas molecules is also important to enhance the catalytic activity^17^,^18^. 3DOM wall structure is in favor of soot transmission while it has disadvantage for adsorption of small gas molecules. Herein supporting microporous structure materials on the 3DOM wall structure could enhance the catalytic activity by macroporous effect for soot transmission and microporous effect for adsorption and activation of small gas molecules. Recently, researchers demonstrated that cryptomelane-type manganese oxide (e.g., manganese oxide octahedral molecular sieve (OMS-2)) has a microporous structure (0.46 nm) arising from edge sharing of 2 × 2 [MnO_6_] octahedral chains to form one-dimensional tunnel structures^19^. More importantly, OMS-2 have mixed valence states of Mn^4+^, Mn^3+^ and/or a small amount of Mn^2+^ sites. The manganese oxides with multiple Mn oxidation states have been reported as promising catalysts for oxidation reactions^20^. In addition, potassium (K)-containing materials, which can increase chemisorbed oxygen, form eutectic compounds and carbonate intermediates, show better catalytic performance for soot combustion than other alkali elements^21^,^22^. However, the catalytic activities of K-based catalysts usually tend to degrade after repeated thermal cycles owing to the loss of potassium. Incorporation of K into a more stable structure is one of effective approaches^23^,^24^. Many K-doped catalysts, including K-doped perovskite catalysts^25^, and K-doped single metal oxides^26^,^27^, have been studied for soot combustion. Because the tunnel cavity of OMS-2 is as large as 0.46 nm, some large cations, such as K^+^, Na^+^, Ba^2+^ and others, are inevitably introduced into the tunnel and stabilized in the catalytic reaction^28^. More interestingly, the K^+^ ion plays an important role in templating and stabilizing the tunneled framework of OMS-2^29^. Due to changing valence states of Mn species in the OMS-2 and high catalytic activity of K for soot combustion, microporous K-OMS-2, which are formed nanoparticles and supported on 3DOM SiO_2_, are expected to enhance catalytic performance for soot combustion by macroporous effect, microporous effect, nano-effect and synergistic effect between K and Mn.

In this paper, a series of novel oxide catalysts, which contain 3DOM structure in the SiO_2_ support and microporous structure in the nano-K-OMS-2, were firstly designed and prepared by a very simple method. The as-prepared catalysts exhibit super high catalytic activities for soot combustions owing to macroporous structure for soot transmission and micropore structure for the activation of small molecules (NO, O_2_). More interestingly, the as-prepared catalysts adopt cheap KNO_3_ and Mn(NO_3_)_2_ materials as active components, thus they cost much lower than noble metal catalysts. They show similar or even higher catalytic activities in medium temperature rage. In addition, the influence of NO and stability of as-prepared catalysts were also studied. The possible mechanisms of catalysts for soot combustion are discussed based on the results of characterization.

## Results

### Structural Features of As-prepared Catalysts

**Figure 1:**
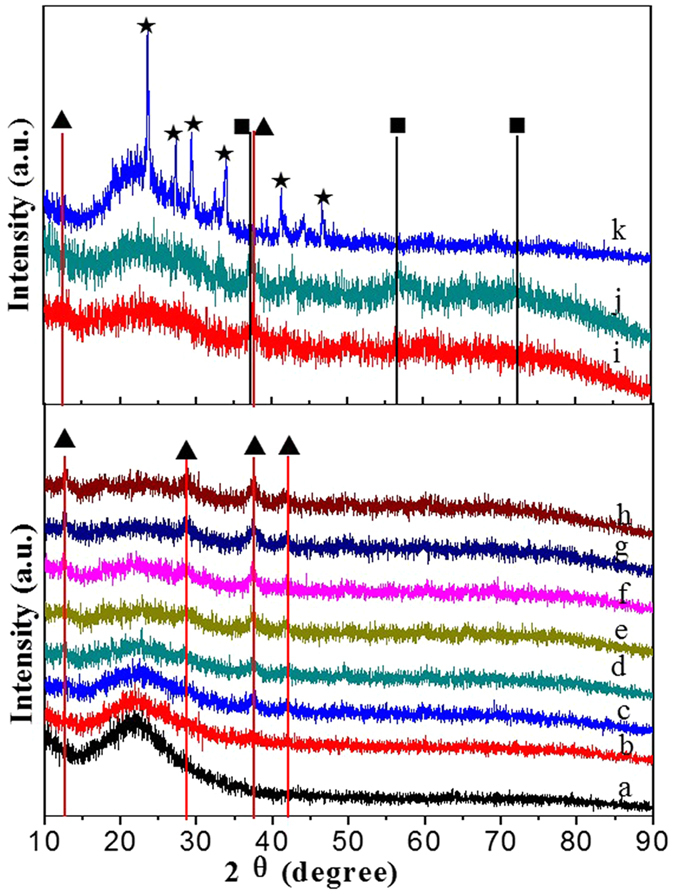
XRD patterns of as-prepared catalysts. SiO_2_ (**a**); K-OMS-2/SiO_2_-10 (**b**); K-OMS-2/SiO_2_-20 (**c**); K-OMS-2/SiO_2_-30 (**d**); K-OMS-2/SiO_2_-40 (**e**); K-OMS-2/SiO_2_-50 (**f**); K-OMS-2/SiO_2_-60 (**g**); K-OMS-2/SiO_2_-70 (**h**); K-OMS-2/Silica gel-50 (**i**); MnO_x_/SiO_2_-50 (**j**); KNO_3_/SiO_2_-50 (**k**).

The XRD patterns results of as-prepared catalysts are shown in the Fig. 1. The Fig. 1a shows the diffraction peaks of pure SiO_2_, which the 23.5° is corresponding to the amorphous SiO_2_. As shown in Fig. 1b–h, the feature peaks, which 2θ degree are located at 12.6°, 28.6°, 37.4°, 41.6°, are corresponding to cryptomelane-M (OMS-2) with a space group of I2/m(12) (JCPDS Card No. 44-1386) and they increased with increasing of K-OMS-2 loading amounts (as “▲” marked in the Fig. 1b–h). Figure 1i suggests that the cryptomelane-M is also synthesized on the surface of silica gel support. Different active components supported on 3DOM SiO_2_ show various feature peaks. The feature peaks (as “■” marked in the Fig. 1j) could be readily indexed to manganese oxides^13^. Those feature peaks, which marked by the “★” in the Fig. 1k, are corresponding to the diffraction peaks of KNO_3_. Different XRD patterns of as-prepared catalysts indicate that the potassium has been doped into the tunnel structure of OMS-2. The above results illustrate that we have successfully prepared a novel ordered micro/macro catalyst, which contains 3DOM SiO_2_ and microporous structure in K-OMS-2 NPs.

**Figure 2:**
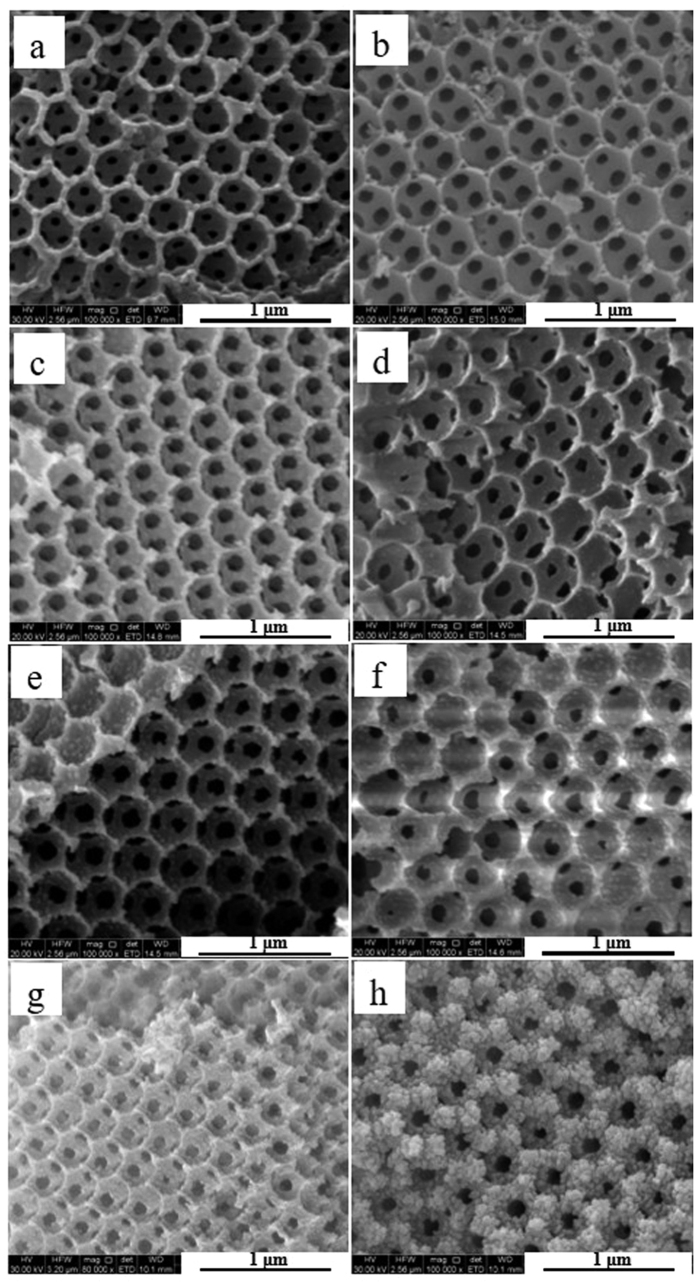
SEM images of 3DOM K-OMS-2/SiO_2_. SiO_2_ (**a**); K-OMS-2/SiO_2_-10 (**b**); K-OMS-2/SiO_2_-20 (**c**); K-OMS-2/SiO_2_-30 (**d**); K-OMS-2/SiO_2_-40 (**e**); K-OMS-2/SiO_2_-50 (**f**); K-OMS-2/SiO_2_-60 (**g**); K-OMS-2/SiO_2_-70 (**h**).

The SEM images (Fig. 2) clearly demonstrate that all 3DOM K-OMS-2/SiO_2_ catalysts have long range ordered macroporous with average diameter of ca. 310 ± 20 nm. As shown in the Fig. 2b–h, more and more visible nanoparticles can be obtained on the skeleton of 3DOM SiO_2_ when the loadings of K-OMS-2 NPs are increased. Especially, when the loading of K-OMS-2 is higher than a certain value (K-OMS-2/SiO2–60), the surface of 3DOM SiO_2_ is completely covered by K-OMS-2 NPs and the pores of 3DOM SiO_2_ were also filled (Fig. 2h). Combined with the XRD results (Fig. 1a–h), the K-OMS-2 NPs, which supported on the surface of 3DOM SiO_2_, would be contributed to enhancing the intensity of diffraction peaks for cryptomelane-M. In order to compare the influence of different active components and supports, the SEM images of K-OMS-2/silica gel, MnO_x_/SiO_2_-50 and KNO_3_/SiO_2_-50 are also exhibited in the Supporting information. As shown in the Fig. S2, K-OMS-2/silica gel is constituted by nanoparticles (Fig. S2a). SEM images of MnO_x_/SiO_2_-50 and KNO_3_/SiO_2_-50 (Fig. S2b and c) indicate that 3DOM SiO_2_ is stable when different active components supported on the surface of 3DOM structure.

**Figure 3:**
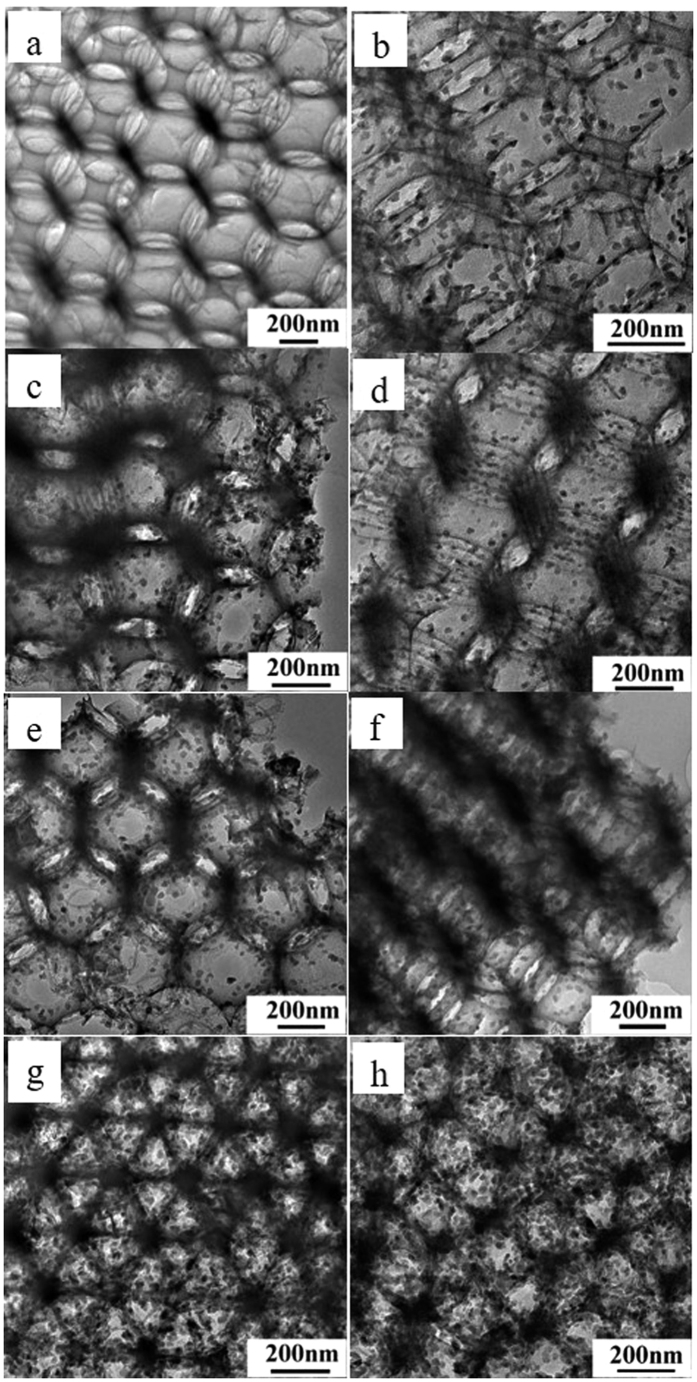
TEM images of 3DOM K-OMS-2/SiO_2_ catalysts. SiO_2_ (**a**); K-OMS-2/SiO_2_-10 (**b**); K-OMS-2/SiO_2_-20 (**c**); K-OMS-2/SiO_2_-30 (**d**); K-OMS-2/SiO_2_-40 (**e**); K-OMS-2/SiO_2_-50 (**f**); K-OMS-2/SiO_2_-60 (**g**); K-OMS-2/SiO_2_-70 (**h**).

TEM images of 3DOM K-OMS-2/SiO_2_ catalysts are shown in the Fig. 3. From the Fig. 3a, no any K-OMS-2 NPs are covered on the skeleton of 3DOM SiO_2_. However, as shown in Fig. 3b–h, the surface of 3DOM SiO_2_ is successfully decorated with well-dispersed K-OMS-2 NPs and no larger agglomerated particles is observed. The average K-OMS-2 NPs sizes are estimated to be 20–25 nm for 3DOM K-OMS-2/SiO_2_ catalysts with different K-OMS-2 loadings (see in the Fig. S5). The densities of K-OMS-2 NPs on the skeleton of 3DOM SiO_2_ are increased with the enhancement of loading dosages of active components. TEM images of Fig. 3g and h exhibit that the surface of 3DOM SiO_2_ is completely covered by K-OMS-2 NPs, which is agreed with the SEM results (Fig. 2). Figure S3 shows TEM images of catalysts with different supports and active components. It indicates that the silica gel is composed of SiO_2_ particles, and no obvious particles of K-OMS-2 are observed on the silica gel (Fig. S3a). The reason for this phenomenon is that the precursor solution of KNO_3_ and Mn(NO_3_)_2_ is infiltrated into the inner of silica gel through accumulation pores. Figure S3b shows the TEM image of MnOx/SiO_2_-50. The surface of 3DOM SiO_2_ is covered by nanoparticles of MnO_x_, which the particle size is about 22 nm (Fig. S5h). As shown in the Fig. S3c, no particles of KNO_3_ appeared on the surface of 3DOM SiO_2_.

**Figure 4:**
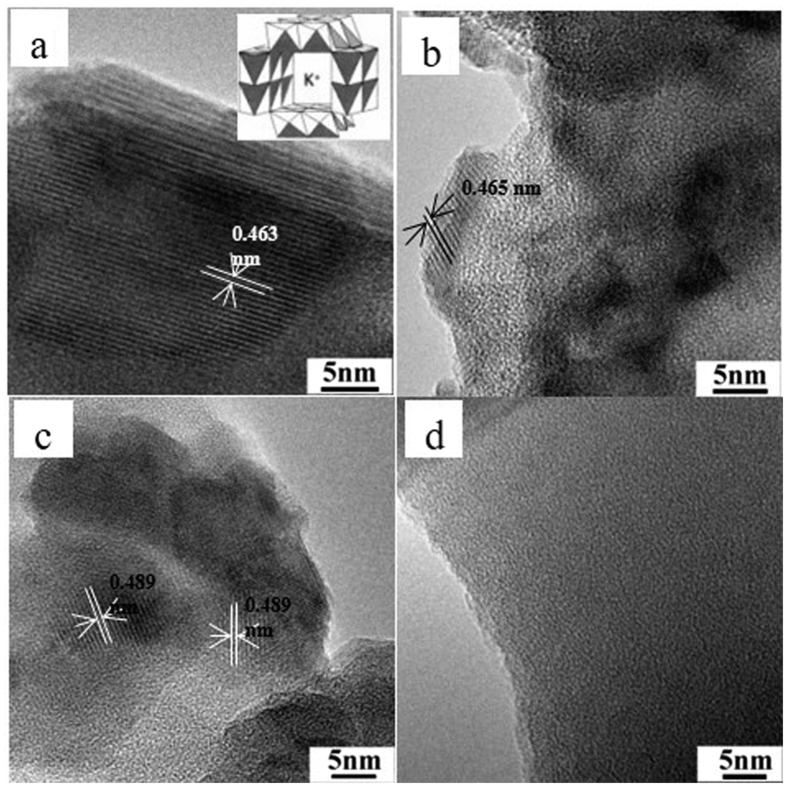
HRTEM images of K-OMS-2/SiO_2_-50 (**a**), K-OMS-2/Silica gel-50 (**b**), MnO_x_/SiO_2_-50 (**c**) and KNO_3_/SiO_2_-50 (**d**).

The HRTEM images of as-prepared catalysts with different supports and active components are shown in Fig. 4. HRTEM image of K-OMS-2/SiO_2_-50 shows that the lattice fringes of K-OMS-2 NPs are clearly observed (Fig. 4a), e.g. the interplanar spacing is measured to be 0.463 nm indexed as (002) planes of K-OMS-2. As shown in the Fig. S4a–g, clear lattice fringes can be observed on the K-OMS-2/SiO_2_ with various K-OMS-2 loadings. The lattice fringes are corresponding to the crystal of K-OMS-2. Those results indicate that K-OMS-2 nanoparticles contain a microporous structure (0.46 nm) arising from edge sharing of 2 × 2 [MnO_6_] octahedral chains to form one-dimensional tunnel structures (as shown insert of Fig. 4a)^20^. Because the tunnel cavity of OMS-2 is as large as 0.46 nm, the K^+^ cations are inevitably introduced into the tunnel and stabilized in the catalytic reaction. From the Fig. 4b, the irregular K-OMS-2 particles are appeared on the surface and inner of silica gel. And the lattice fringes was measured to be 0.465 nm indexed as (002) planes of K-OMS-2. As shown in the Fig. 4c, the distinct lattice fringes (0.489 nm) are assigned to the (200) planes of manganese oxides. Figure 4d shows an interesting result of KNO_3_/SiO_2_-50, no particles or sheets of KNO_3_ can be observed on the surface of SiO_2_, indicating that the KNO_3_ may be adhered on the surface of SiO_2_ with molten state during the calcined process. After finishing of calcined process, the KNO_3_ are uniform distributed on the skeleton of SiO_2_.

**Figure 5:**
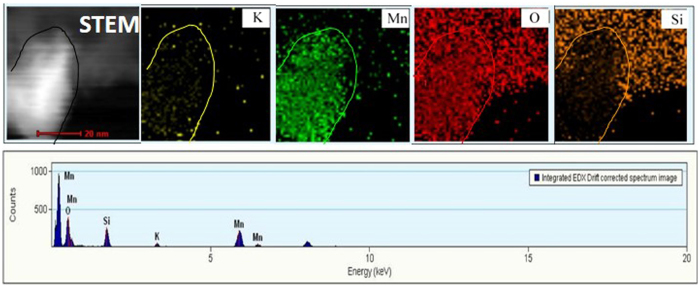
HAADF-STEM image and EDS elemental mapping of K-OMS-2/SiO_2_-50.

In order to get the distributed information of K, Mn, O and Si elements in the as-prepared catalysts, the HAADF-STEM image and EDS elemental mapping of K-OMS-2/SiO_2_-50 are shown in the Fig. 5. The EDX elemental mappings suggest that the O and Si are uniformly distributed in the in the αorts of 3DOM SiO_2_. Due to O element in the K-OMS-2 NPs, the O element can be found more riches in the K-OMS-2 NPs while the Si element cannot be obviously observed in the K-OMS-2 NPs. As shown in the Fig. 5, the Mn element is aggregated in the K-OMS-2 NPs owing to Mn is the main components for K-OMS-2 NPs. In addition, the K element is also focused on the K-OMS-2 NPs. However, the intensity of K element is seemed weaker than corresponding that of Mn element. The possible reason for this phenomenon is that the K elements are doped into the one-dimensional tunnel structures of 2 × 2 [MnO_6_] octahedral chains.

### TGA-DSC Analyses of As-prepared Catalysts

**Figure 6:**
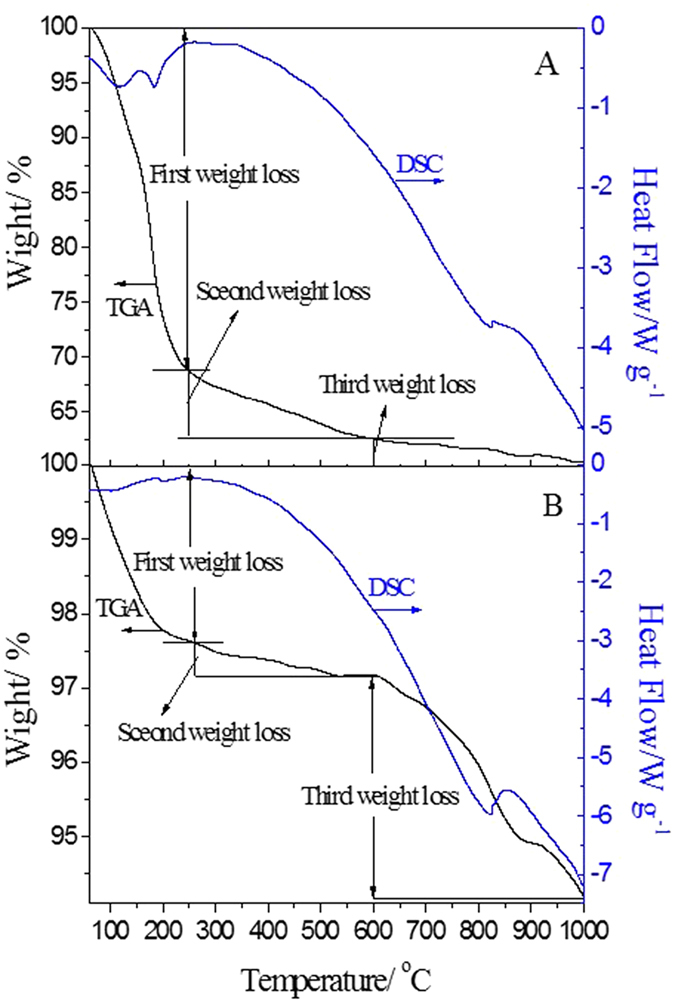
TG-DSC curves of K-OMS-2/SiO_2_-50. (**A**) none calcined, (**B**) calcined.

The weight loss and heat flow of K-OMS-2/SiO_2_-50 samples (none calcined and calcined) observed during thermogravimetry-differential thermal analyses (TGA-DSC) in an oxygen atmosphere are displayed in Fig. 6. As shown by the TGA curve of none calcined K-OMS-2/SiO_2_-50 (Fig. 6A), weight losses occurred in three temperature ranges of 50–250, 250–600 and 600–1000 °C. The first significant weight loss of 31.3% is mainly attributed to the decomposition of KNO_3_ and Mn(NO_3_)_2_. In this process, the K and Mn ions are formed impermanent species of K-OMS-2, so that the NO_3_^−^ in the KNO_3_ is separated and decomposed. Certainly, desorption of physisorbed and chemisorbed water is also contributed to the first significant weight loss. The second significant weight loss of 4.2% in the temperature range of 250–600 °C is generally due to further dehydration, lattice oxygen loss or part nitrate decomposition of K-OMS-2. The third slight weight loss of 2.1% is started at 600 °C and ended at 1000 °C. In this temperature range, the collapse and the change of K-OMS-2 to hausmannite by the transformation of Mn^4+^ to Mn^3+^ are the possible reasons for lattice oxygen release^30^. As you can see from the DSC curve of none calcined K-OMS-2/SiO_2_-50, a small exothermic peak appeared in the range of 120–180 °C with the highest exothermic value at ca. 160 °C. This exothermic peak reveals the thermal decomposition of nitrate, which came from the preparation process. With increasing of temperature, as a result of dehydration and crystal phase transformation, an endothermic process appears in the DSC curve when the temperature is over 250 °C. Another small exothermic peak at 830 °C is corresponding to crystal change of K-OMS-2^31^. Figure 6B shows the TGA-DSC curves of calcined K-OMS-2/SiO_2_-50. Compared with TG curve of none calcined K-OMS-2/SiO_2_-50, the calcined K-OMS-2/SiO_2_-50 has less weight loss owing to decomposition of nitrate in the calcination process. The weight loss also could be divided into three parts in the range of 50-1000 °C. The temperatures of the first weight loss are in the range of 50–250 °C. Because the nitrate has been decomposed in the calcination process, the first weight loss about 2.4% can be attributed to desorption of physisorbed and chemisorbed water. The second weight loss is occurred in the temperature range of 250–600 °C and the ratio of weight loss is 0.5%. Similarly, further dehydration and lattice oxygen loss of K-OMS-2 may be contributed to this weight loss. The third weight loss is 2.9% and temperature range is 600–1000 °C^32^. The reasons for this weight loss are similar to that of none calcined K-OMS-2/SiO_2_-50. As shown in Fig. 6B, compared with DSC curve in the Fig. 6A, the DSC curve of calcined K-OMS-2/SiO_2_-50 indicates that no exothermic peak appears in the range of 120–180 °C due to no nitrates in the calcined sample. However, the curve of calcined K-OMS-2/SiO_2_-50 is the same as that of none calcined K-OMS-2/SiO_2_-50 when the temperature is over 250 °C. An overview of TG-DSC characterizations indicates that the as-prepared catalysts are stable under the calcination temperature of 600 °C. If the calcination temperature is over 600 °C, the crystal form of K-OMS-2 would be changed. The content of exchangeable active oxygen in the range of 250–600 °C may affect the physicochemical performances of as-prepared catalysts.

### Raman spectra of As-prepared Catalysts

**Figure 7:**
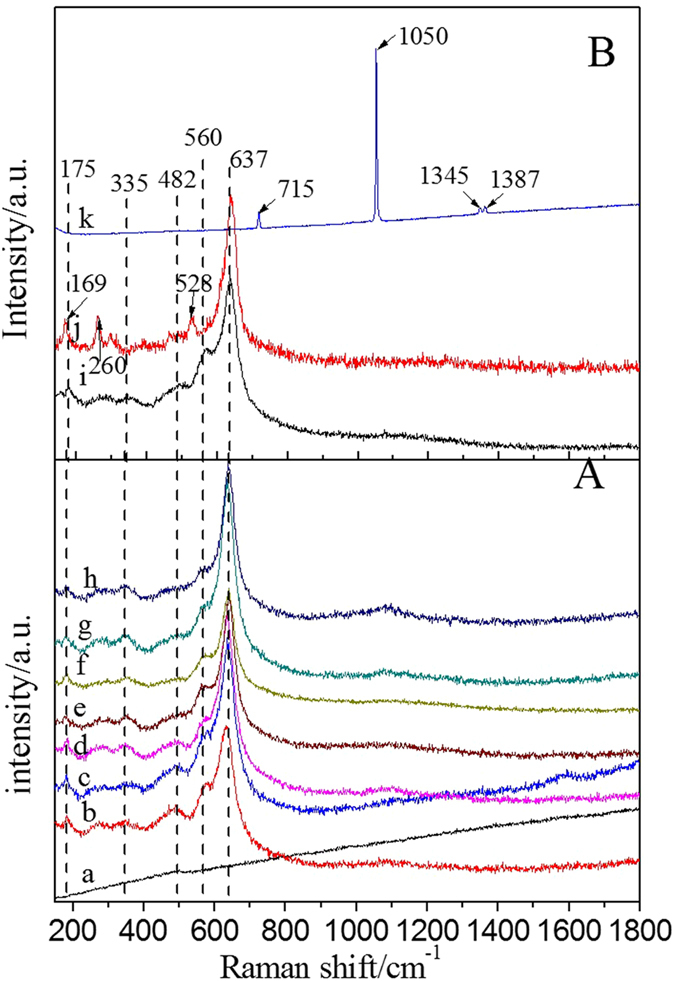
Raman spectra of as-prepared catalysts. (**A**) K-OMS-2/SiO_2_ obtained at varied K-OMS-2 loadings: SiO_2_ (a), K-OMS-2/SiO_2_-10 (b), K-OMS-2/SiO_2_-20 (**c**), K-OMS-2/SiO_2_-30 (d), K-OMS-2/SiO_2_-40 (e), K-OMS-2/SiO_2_-50 (f), K-OMS-2/SiO_2_-60 (g) and K-OMS-2/SiO_2_-70 (h) (**B**) different support and active components on the 3DOMSiO_2_: K-OMS-2/silica gel-50 (i), MnOx/SiO_2_-50 (j) and KNO_3_/SiO_2_-50 (k).

In an effort to investigate the influence of K doping on the microscopic structure and vibrational properties of MnO_x_, Raman spectra of the K-OMS-2/SiO_2_ with different K-OMS-2 loading amounts and 3DOM SiO_2_ with different active components were measured, and the results are shown in Fig. 7. For the pure 3DOM SiO_2_ (Fig. 7a), a weak Raman peak at about 492 cm^−1^ is observed, which is corresponding to the vibration of Si-O^33^. When the K-OMS-2 was loaded on the 3DOM SiO_2_, five Raman peaks, including 637, 560, 482, 335 and 175 cm^−1^, are observed in the Raman shift range of 150–1000 cm^−1^ 34. The Raman peaks are maintained regular locations with increasing of K-OMS-2 loading. The peaks at 175 and 335 cm^−1^ can be ascribed to the deformation modes of Mn-O-Mn, while the peaks at 637 and 560 cm^−1^ can be assigned to the Mn-O vibrations that are orthogonal and along the direction of the MnO_6_ octahedral double chains^35^,^36^. The peak at 482 cm^−1^ (Raman shift of SiO_2_) disappears when the mass ratio of KNO_3_ and SiO_2_ is exceeded 20%. This result indicates that the surface of SiO_2_ is covered by K-OMS-2 nanoparticles, which agrees well with the results of SEM and TEM.

As shown in Fig. 7i, the Raman peaks of K-OMS-2/silica gel-50 are similar to that of K-OMS-2/SiO_2_-50. Figure 7j shows Raman spectrum of MnO_x_/SiO_2_-50. Four bands can be observed at 639, 528, 260 and 169 cm^−1^ and they are assigned to Mn-O and Mn-O-Mn stretching mode of MnO_x_, respectively. The MnO_2_ and Mn_2_O_3_ components are contributed to the most intense Raman peak at 639 cm^−1^ 37. Compared with the K-OMS-2/SiO_2_, the Raman peak of Mn-O-Mn shifts to low wavenumber due to no influence of other ions. The Raman shifts of as-prepared catalysts in the Raman spectra demonstrate the doping of K in the OMS-2, this phenomenon also demonstrated by the XRD results (Fig. 1). The Raman peaks of KNO_3_/SiO_2_-50 are located at 715, 1050, 1345 and 1387 cm^−1^. The 715 and 1050 cm^−1^ are belonged to the totally symmetric ν_1_ and the doubly degenerate ν_4_ modes of NO_3_^−^, respectively. The obvious difference in Raman peaks of KNO_3_/SiO_2_-50 and K-OMS-2/SiO_2_-50 suggest that K ions have completely doped into the MnO_x_ and formed complex K-Mn oxides. The Raman spectra indicate that the weak Mn-O bands are obtained in the K-OMS-2 nanoparticles due to doping of K^+^. Surface weak Mn-O bands are proposed to take part in activation of oxygen more easily. Therefore, the K-OMS-2/SiO_2_ catalyst may show high catalytic activity than that of MnO_x_/SiO_2_.

### H_2_-TPR Results of As-prepared Catalysts

**Figure 8:**
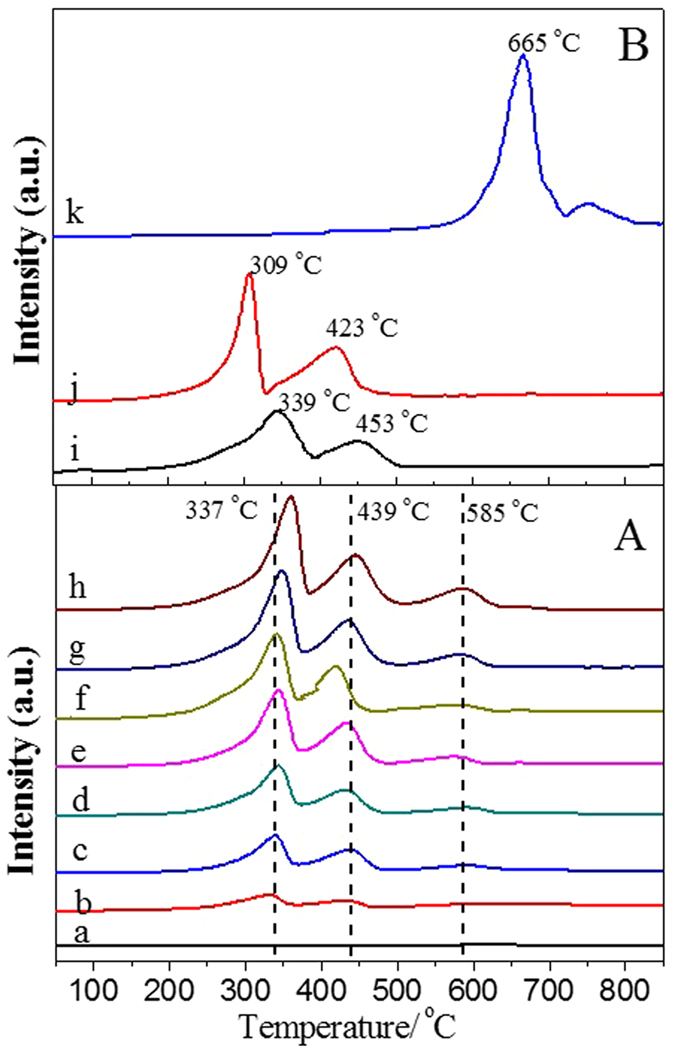
H_2_-TPR curves of as-prepared catalysts. (**A**) K-OMS-2/SiO_2_ obtained at varied K-OMS-2 loadings: SiO_2_ (a), K-OMS-2/SiO_2_-10 (b), K-OMS-2/SiO_2_-20 (c), K-OMS-2/SiO_2_-30 (d), K-OMS-2/SiO_2_-40 (e), K-OMS-2/SiO_2_-50 (f), K-OMS-2/SiO_2_-60 (g) and K-OMS-2/SiO_2_-70 (h); (**B**) different support and active components on the 3DOMSiO_2_: K-OMS-2/silica gel-50 (i), MnO_x_/SiO_2_-50 (j) and KNO_3_/SiO_2_-50 (k).

Catalytic combustion of soot is a complicated gas-solid (soot)-solid (catalyst) multi-phase reaction. The intrinsic redox properties of catalysts play a key role in the combustion of soot. Therefore, temperature-programmed reduction (TPR) by H_2_ was used to measure these characteristics in the present work. As shown in Fig. 8, the peak position and types are similar for 3DOM K-OMS-2/SiO_2_ with different K-OMS-2 loadings, while the intensity of H_2_ consumption increases with the increasing of K-OMS-2 loading. For 3DOM SiO_2_ (Fig. 8a), no reduction peak is observed. In the H_2_-TPR profiles of 3DOM K-OMS-2/SiO_2_ catalysts, there are three overlapping peaks (ranging from 250 to 700 °C), corresponding to a three-step reduction process. Reduction peaks are observed in three temperature ranges of 250–380 °C, 380–500 °C and 500–650 °C. Assuming that MnO is the final state in the reduction of OMS-2 Mn species^38^. The peak at 200–350 °C could be assigned to the reduction of MnO_2_/Mn_2_O_3_ to Mn_3_O_4_, and the peak at 380–500 °C may be assigned to the reduction of Mn_3_O_4_ to MnO. The results indicate that substantial amount of Mn^4+^ and Mn^3+^ in K-OMS-2 can be reduced to Mn^2+^ below 500 °C, which is consistent with the previous reports^39^. The third reduction peak at 500–650 °C may be assigned to the reduction of K species. When the mass ratio of KNO_3_ to SiO_2_ is over 25%, the intensity of K species reduction peak is obviously observed, and increases with increasing of K-OMS-2 loading amounts. The H_2_-TPR profiles of K-OMS-2/silica gel, 3DOM MnO_x_/SiO_2_–50 and KNO_3_/SiO_2_ catalysts are presented in Fig. 8B. The peak position and types vary among the catalysts indicate different redox performance of as-prepared catalysts. Because of poor redox performance of SiO_2_ supports and the same active components, the reduction peaks of K-OMS-2/silica gel-50 (Fig. 8i) are similar to that of 3DOM K-OMS-2/SiO_2_-50. As for MnO_x_/SiO_2_-50, there are two main peaks at 309 °C and 423 °C, while no reduction peak is observed at relatively high temperature. It is apparent that the TPR peaks of 3DOM MnO_x_/SiO_2_-50 are present at lower temperature, indicating higher reducibility of manganese in MnO_x_ than the corresponding 3DOM K-OMS-2/SiO_2_-50. The TPR profile of KNO_3_/SiO_2_-50 catalyst presents two important reduction signals at 530–720 °C and 720–830 °C assigned to the potassium nitrate reduction. The potassium nitrate can be reduced with H_2_ to generate KNO_2_, NO and NH_3_. The presence of the H_2_ consumption peak for KNO_3_/SiO_2_-50 indicates the presence of main KNO_3_ species on the surface of 3DOM SiO_2_ even after calcination at 550 °C. It is in good agreement with the XRD results of 3DOM KNO_3_/SiO_2_-50 (Fig. 1k).

### XPS spectra of As-prepared Catalysts

**Figure 9:**
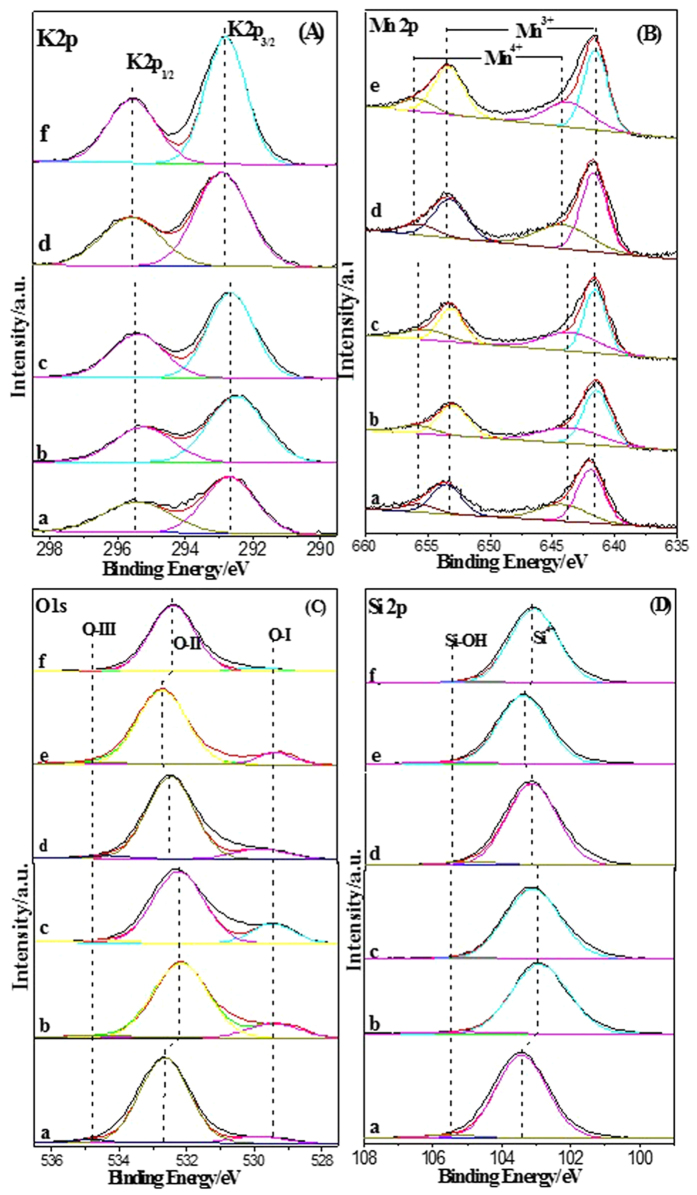
K 2p (**A**), Mn 2p (**B**), O1s (**C**) and Si 2p (**D**) XPS spectra of as-prepared catalysts (K-OMS-2/SiO_2_-20 (a), K-OMS-2/SiO_2_-50 (b), K-OMS-2/SiO_2_-70 (c), K-OMS-2/silica gel-50 (d), MnOx/SiO_2_-50 (e) and KNO_3_/SiO_2_-50 (f)).

XPS studies are conducted to gain insight into the oxidation state, surface composition, and atomic environment of K, Mn, O and Si species in the different samples, and the results are shown in Fig. 9 and Table 1. As shown in Fig. 9A, the K 2p spectrum consists of a spin-orbit split doublet composed of two peaks with an intensity ratio between K 2p_3/2_ and K 2p_1/2_ at about 2:1. The binding energies (BEs) of 292.4–292.8 eV and 295.2–295.6 eV are assigned to K 2p_3/2_ and K 2p_1/2_ (Table S2), respectively, which the separation of K 2p_3/2_ and K 2p_1/2_ peaks is about 2.6 eV^40^. The various binding energies of K indicates that environment of K is different in the as-prepared catalysts. Figure 9B displays the Mn 2p XPS of as-prepared catalysts. The Mn 2p spectra are significantly broadened and show some asymmetry towards both Mn 2p_3/2_ and Mn 2p_1/2_ peaks. Due to this feature, it is worth noting that the binding energies of components of as-prepared catalysts are in good agreement with the literature data reported for Mn^3+^ and Mn^4+^ 41. As shown in Fig. 9B, the binding energies of the XPS Mn 2p_3/2_ peaks are found to be in the range 640.0–645.0 eV. Two kinds of Mn species including Mn^3+^ (ca. 641.5 eV), and Mn^4+^ (644.5 eV) are presented on the surface of as-prepared catalysts. Meanwhile, the Mn 2P_1/2_ peak also shows two kinds of Mn species in the BEs range of 650–655 eV (Table S2). The detailed peak fitting results of Mn 2p features for the two Mn ions are listed in Table 1. Apparently, the K-OMS-2 loadings have a slight impact on the surface Mn^3+^/Mn^4+^ molar ratio. Table S2 indicates that the binding energies of Mn element decreased with increasing of K-OMS-2 loadings. The small change of BEs of Mn indicates the electronic densities of Mn atoms increase, suggesting that the low-valence Mn species increased^42^. Quantitative analysis of the surface components also proves that Mn species in the high loading of K-OMS-2 have lower average oxidation state than that of low loading catalyst^43^. Among those catalysts, because of the same K-OMS-2 loadings, the K-OMS-2/SiO_2_-50 and K-OMS-2/silica gel-50 show similar surface Mn^3+^/Mn^4+^ molar ratio (1.93 and 1.97, respectively), whereas MnO_x_/SiO_2_-50 is the lowest in terms of surface Mn^3+^/Mn^4+^ molar ratio (1.65). More Mn^3+^ sites may be originated a weak Mn-O bond and formed more active oxygen species, which enhanced catalytic activity for redox reaction^42^.

The corresponding spectra of oxygen species are presented in Fig. 9C. Three types of oxygen species, which are defined as O-I, O-II and O-III, can be observed from the XPS of O1s. The O-I component located at 529.5 eV are ascribed to lattice oxygen ions bonded to metal cations^44^. As shown in Fig. 9a–c, the type of O-I may be considered as mainly generated from Mn-O, K-O-Mn or some Si-O-Mn bonds in the K-OMS-2/SiO_2_. The relative intensity of O-I increases with the increasing of K-OMS-2 loading and the content of O-I increases from 0.073 to 0.173. The high content of O-I indicates that high loading of K-OMS-2 might produce more active oxygen species and show higher catalytic activity. Compared with the K-OMS-2/SiO_2_-50, K-OMS-2/silica gel-50 shows somewhat lower content of O-I owing to parts of K-OMS-2 infiltrated into the inner of silica gel. Due to pure manganese oxide in the MnO_x_/SiO_2_-50, the BE of O-I has a small shift from 529.5 to 529.2 eV. The O-I content of MnO_x_/SiO_2_-50 is similar to that of K-OMS-2/silica gel-50. Because of no thermal decomposition of KNO_3_, the KNO_3_/SiO_2_-50 shows lowest content of O-I and it occupies only 3.7%. The dominating O-II component at BE ~532.5 eV is obviously related to O^2−^ species in SiO_2_ (Si-O-Si environments)^45^. The content of O-II decreases with increasing of K-OMS-2 loading due to more K-OMS-2 NPs on the surface of 3DOM SiO_2_ at the high loading of K-OMS-2. Obviously, the O-II contents of as-prepared catalysts are related to the O-I, in other words, high O-I content is corresponding to low O-II. The minor O-III component located at ~534.6 eV may include contributions from Si-OH groups or adsorbed H_2_O. In addition, in view of the catalyst composition, contribution of −C = O groups (some adsorbent CO_2_ is formed carbonate by K or Mn) is considered to be available. The KNO_3_/SiO_2_-50 shows the highest content of O-III, which is in good agreement with the results of Raman spectra. The Si 2p spectra of the catalysts (Fig. 9D) are dominated by a peak at BE~103.4 eV, characteristic of Si^4+^ in SiO_2_; a smaller component at BE~105.5 eV indicates the presence of Si-OH species^46^.

### Catalytic Performances for Soot Combustion

The catalytic performances of 3DOM K-OMS-2/SiO_2_ catalysts for soot oxidation were evaluated and the results are listed in Table 2. For comparison, the soot combustion reactions of without catalyst and over 3DOM SiO_2_ were also estimated under the same reaction conditions. For the pure soot, the T_10_, T_50_ and T_90_ are 482, 564 and 609 °C, respectively. 3DOM SiO_2_ also shows somewhat catalytic activity for soot combustion, and the T_10_, T_50_ and T_90_ are 354, 503 and 550 °C, respectively. This result indicates that the 3DOM structure of SiO_2_ may enhance the contact area between soot and reaction gas. 3DOM K-OMS-2/SiO_2_ catalysts show high catalytic activities for soot combustion. The catalytic activity enhanced with increasing of K-OMS-2 loading and reached maximum at a certain value (K-OMS-2/SiO_2_-50). Further increasing the K-OMS-2 loadings, the catalytic activity almost keep constant. Compared the ΔT_10_, ΔT_50_ and ΔT_90_ of K-OMS-2/SiO_2_ catalysts in the Table 2, the ΔT_10_ of 3DOM K-OMS-2/SiO_2_-50 catalyst is 199 °C, which is the highest among the 3DOM K-OMS-2/SiO_2_ catalysts (i.e. the K-OMS-2/SiO_2_-50 catalyst has the lowest initiation temperature). As shown in Table 2, the temperature of T_90_ decreases with increasing mass ratio of KNO_3_ and SiO_2_ and it is lower than 400 °C when the K loading is reached a certain value (K-OMS-2/SiO_2_-30). Because of temperature range of 175–400 °C for diesel exhaust, soot particles over the K-OMS-2/SiO_2_ catalysts can be completely burnt off when the diesel engines are normal running (i.e. the temperature of diesel exhaust is ca. 400 °C). In addition, the K-OMS-2/SiO_2_ catalysts show higher CO_2_ selectivity for soot combustion than that of pure soot combustion. All surpassed 90%. The selectivity to CO_2_ increased with increasing of K-OMS-2 loading and the CO_2_ selectivity value of K-OMS-2/SiO_2_-50 catalyst is as high as 96.7%. In order to deeply investigate the interaction effect (K and Mn) and macropore effect for soot combustion, the catalytic activities of K-OMS-2/silica gel-50, 3DOM MnO_x_/SiO_2_-50 and 3DOM KNO_3_/SiO_2_-50 were tested and the results are shown in Table 2. Compared with 3DOM K-OMS-2/SiO_2_-50, K-OMS-2/silica gel-50 shows much lower catalytic activity indicating that the 3DOM structure is favorable for soot combustion. It is attributed to that K-OMS-2 may be infiltrated into the inner of silica gel through accumulation porous (see the TEM image of Fig. S3), so that most of K-OMS-2 contact soot particle more difficultly than 3DOM K-OMS-2/SiO_2_-50. 3DOM MnO_x_/SiO_2_-50 and KNO_3_/SiO_2_-50 show higher activity than K-OMS-2/silica gel-50 but lower than 3DOM K-OMS-2/SiO_2_-50, indicating that the interaction effect between K and Mn has positive influence on soot combustion. In order to compare the catalytic performances of 3DOM as-prepared catalysts, the catalytic activities of previous other catalysts reported in literature are summarized in Table 3. When a comparison is made on catalysts for soot combustion, it must be noted that experimental conditions may be deviated from each other concerning reaction gas, catalyst dosage and measurement system. Thus, fine activity comparison of different catalysts is usually challenging. Nevertheless, a rough comparison, including the temperature for soot combustion, is still feasible. As shown in Table 3, the 3DOM K-OMS-2/SiO_2_-50 catalyst showed lower temperature for T_50_ than the previous reported catalysts except the 3DOM Pt_0.08_/Ce_0.8_Zr_0.2_O_2_. From the Table 3, the K-OMS-2/SiO_2_-50 gives the lowest T_90_, i.e., the highest catalytic activity than other 3DOM metal oxides at high reaction temperature (i.e. comparison of T_90_). In addition, the T_90_ of K-OMS-2/SiO_2_-50 is much lower than 400 °C, indicating that the soot can be completely burnt off blew 400 °C. The temperature for complete catalytic combustion of soot is lower than the highest temperature of exhaust (175–400 °C), which is important for practical application. More importantly, the 3DOM K-OMS-2/SiO_2_ catalysts were prepared by simple method and took very cheap KNO_3_ and Mn(NO_3_)_2_ materials as active components. Obviously, non-noble metal catalysts show similar or even higher catalytic activities than noble metal catalysts in the medium and high temperature rage (see the Table 3). However, the as-prepared catalysts show lower catalytic activities than noble metal catalysts in the low temperature rage (temperature of T_10_). The reason for this phenomenon is that the K-OMS-2 NPs have relatively lower redox property than noble metal catalysts for activation of oxygen species at low temperature (<300 °C). When the temperature is over 300 °C, a mass of oxygen can be activated and then the catalytic activities show great enhancement. This result is also well agreed with characterization of H_2_-TPR (Fig. 8). Considering those factors, the catalytic activity of current 3DOM K-OMS-2/SiO_2_ catalysts are significantly enhanced for soot combustion especially K-OMS-2/SiO_2_-50 (T_50_ and T_90_ at ca. 328 °C and 363 °C). Therefore, 3DOM K-OMS-2/SiO_2_ catalysts are promising for the practical application due to high catalytic activity and low cost.

### Catalytic Performance of K-OMS-2/SiO_2_-50 with Different Concentrations of NO in the Reactant Gases

**Figure 10:**
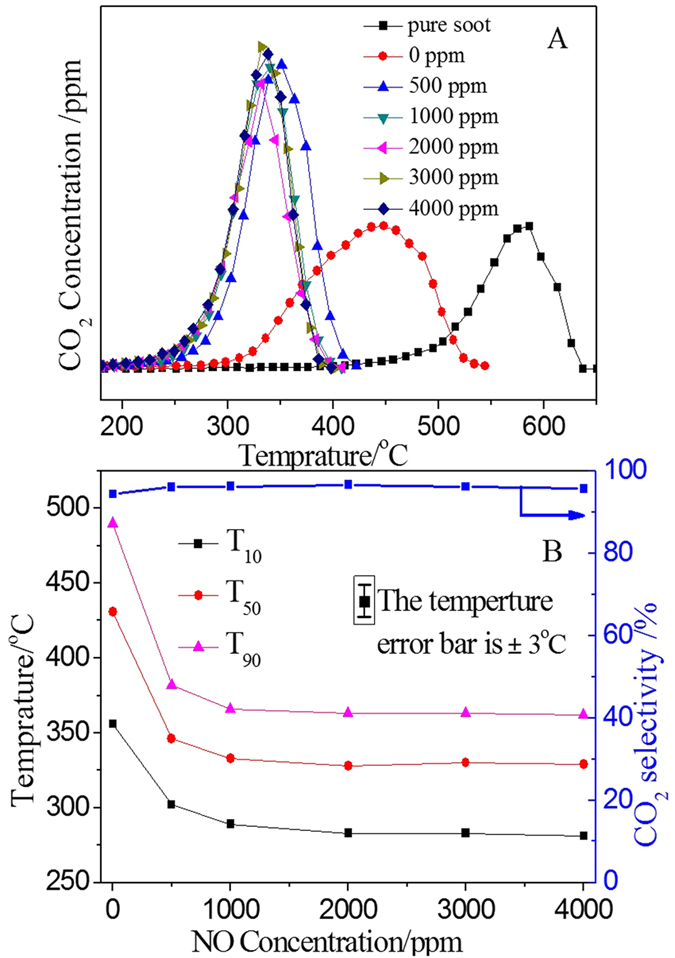
The CO_2_ concentration profiles (**A**) and catalytic activities (**B**) of K-OMS-2/SiO_2_-50 under different concentrations of NO (The temperature error bar is ±3 °C).

In this work, in order to test and research the catalytic performance of as-prepared catalysts more deeply, the catalytic activities of K-OMS-2/SiO_2_-50 with different concentrations of NO were also measured and the results are displayed in the Fig. 10. As shown in Fig. 10A, the profiles of CO_2_ concentration over the K-OMS-2/SiO_2_-50 show different tendencies under the condition of different concentration of NO. In the absence of catalyst, the temperature of highest CO_2_ concentration is located at 580 °C. However, the temperature shifts to 450 °C when catalyst is presented. Moreover, the CO_2_ profiles are higher and narrower than the case without NO. It suggested that the catalytic activities of K-OMS-2/SiO_2_ are strongly affected by the NO gas. In order to more clearly describe the differences of catalytic activities under various concentrations of NO, the T_10_, T_50_, T_90_ and CO_2_ selectivity are calculated and shown in Fig. 10B. The catalytic activity of 3DOM K-OMS-2/SiO_2_-50 for soot combustion increases with increasing of concentration of NO when the NO is lower than 2000 ppm. However, the catalytic activity is almost kept constant when the NO is exceeded 2000 ppm. For comparison, the TPO result without NO is also included in Fig. 10B. Its T_10_, T_50_ and T_90_ are 356, 431 and 490 °C, respectively. The result indicates that NO gas plays an important role in the soot combustion when its concentration is located at low value while high concentration has less assistance. As the same as reported in the previous literature^23^, NO acts as an efficient mobile oxidizing agent, and the NO could be oxidized to NO_2_ by oxygen during the reaction when catalysts are used. Since the oxidizing ability of NO_2_ is stronger than that of O_2_, the removal of soot particles by NO_2_ is one of the main ways in soot combustion. Therefore, the soot combustion subtly changed solid (soot)-solid (catalyst) contact into solid (soot)-gas (NO_2_)-solid (catalyst) contact. This altering reaction path greatly promotes soot combustion so that the catalysts show higher activities than that without NO^16^. More deeply explains will discuss in the following discussion.

### Stability of 3DOM K-OMS-2/SiO_2_-50 Catalyst

**Figure 11:**
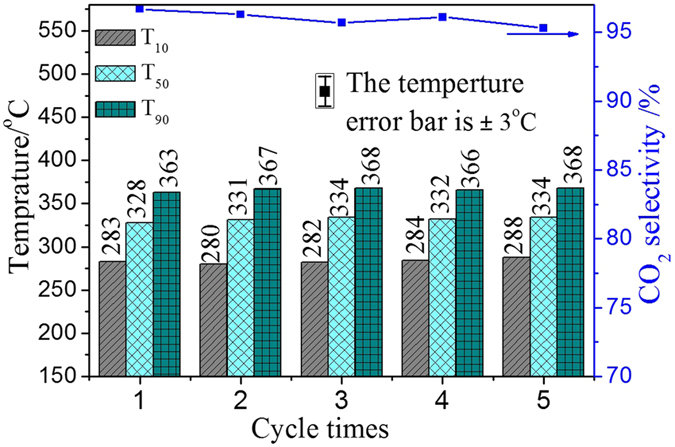
Tested stability results of 3DOM K-OMS-2/SiO_2_-50 catalyst for soot combustion (The temperature error bar is ±3 °C).

**Figure 12:**
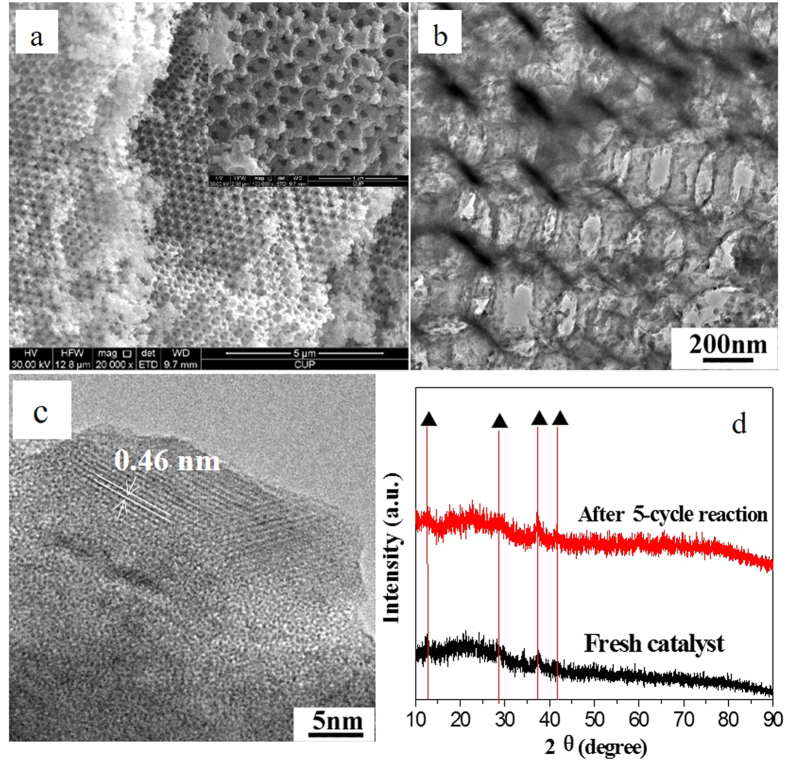
SEM (**a**), TEM images (**b,c**), and XRD (**d**), of 3DOM K-OMS-2/SiO_2_-50 catalyst after 5-cycle reaction.

The stability of catalysts is one of the most important performances, especially in practical application. Therefore, the stability of 3DOM K-OMS-2/SiO_2_-50 catalyst in five cycles running was examined and the results are shown in Fig. 11. 3DOM K-OMS-2/SiO_2_-50 maintained high catalytic activity and CO_2_ selectivity after five-cycle reaction under the condition of loose contact between catalysts and soot particles, the numerical values of T_10_, T_50_ and T_90_ were 283 ± 5, 328 ± 6 and 363 ± 5 °C, respectively. Meanwhile, the CO_2_ selectivity value was more than 95% after reaction for five cycles. In this work, the 3DOM structure of SiO_2_ support and K-OMS-2 nanoparticles play important roles in the catalytic combustion of soot. The used 3DOM K-OMS-2/SiO_2_-50 catalyst was characterized by measurements of SEM, TEM and XRD and the results are listed in the Fig. 12. According to the SEM and TEM images of used 3DOM K-OMS-2/SiO_2_-50 catalyst, 3DOM structure is not destroyed after five cycles reaction, and the mean size (23 ± 6 nm) of K-OMS-2 nanoparticles on the surface of 3DOM SiO_2_ support is not remarkably changed in comparison with that of the fresh sample (24.25 ± 3.37 nm). The XRD similarity between used and fresh catalysts suggests that the crystal form and one-dimensional tunnel structure (0.46 nm) of K-OMS-2 are stable during the process of soot combustion. The characterization results of used catalyst indicate that 3DOM K-OMS-2/SiO_2_ catalysts have good thermal stability and activity stability during soot combustion reaction.

## Discussion

### Macropores Effect of 3DOM SiO_2_

It is well-known that the catalytic combustion of soot is a gas-solid-solid reaction, which the contact between soot and catalysts is one of the most important factors for improving the catalytic performance. In fact, many studies have demonstrated that the tight contact between soot and catalysts shows excellent catalytic performances for soot combustion^23^. However, loose contact between soot particles and catalysts is a typical way of contact in the process of after-treatment for diesel engine exhaust. Therefore, it is extremely important to study and design the active catalysts, which can improve the contact efficiency between the catalysts and soot particles under loose contact conditions. In order to enhance the contact efficiency, 3DOM materials with uniform pore size and periodic voids interconnected through open windows are designed and synthesized by colloidal crystal template method. As shown in Fig. S1a, the average diameter of ordered macroporous is about 310 nm and diameter of interconnected open windows is about 100 nm. Soot particles could easily across those pores and reach the active sites on the inner wall of 3DOM materials with the help of gas-flow Thus, 3DOM structures could enhance the contact between soot particles and catalysts.

**Figure 13:**
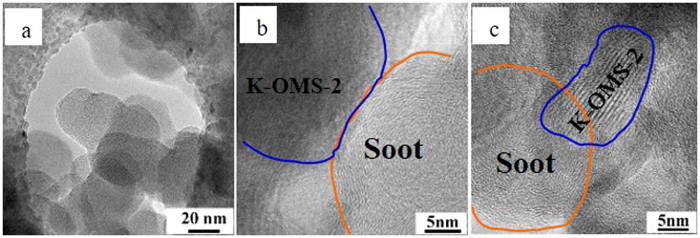
TEM (**a**), and HRTEM (**b,c**), images of 3DOM K-OMS-2/SiO_2_-50 and soot complex. TEM image are obtained at the temperature 280 °C, reaction conditions: 10% O_2_, 2000 ppm NO, Ar balance gas, total gas flow: 50 mL/min.

In this work, in order to demonstrate the contact efficiency, the soot and 3DOM K-OMS-2/SiO_2_-50 catalyst was studied under the same conditions for TPO reaction. In this confirmatory experiment, the reaction temperature of soot and 3DOM catalyst was programmed to 280 °C, which means the soot was not ignited. As we all know, the catalytic soot combustion is a gas-solid-solid reaction, which soot particles can enter the inner pores of 3DOM catalyst with the help of the reaction gas flow (O_2_, NO and Ar) during the reaction process. More importantly, the rising reaction temperature may contribute to separating the agglomerate soot particles. Under the influence of gas flow and rising temperature, the soot particles can easily enter into the 3DOM structure and contact the inner active sites of 3DOM catalyst. As shown in Fig. 13a, the macropores of catalyst contacts with soot particles, indicating that 3DOM structure is a desirable feature for diesel soot combustion. This is the direct evidence that the perfect macroporous structure provides the ideal reaction place to solid reactants (diesel soot). As shown in HRTEM images (Fig. 13b and c), the soot and K-OMS-2 nanoparticles are well contact each other in the inner pores of 3DOM structure. Due to this contact efficiency, the number of available active sites of catalysts can be maximized for utilization, especially inner active sites of catalysts. More active sites would result in higher catalytic activity. The uniform macroporous network allows easy mass transfer and less diffusional resistance when the large size materials such as soot particles go through the catalyst structure. TEM results have intuitionally demonstrated that soot particles can easily enter the interior of 3DOM catalysts with the help of the airflow in the reaction process under the loose contact conditions, and have less resistance to go through the catalyst structure. In fact, our group has synthesized a series of 3DOM materials and they show higher catalytic activities than the corresponding particle materials^10^,^11^,^14^. Therefore, it is significantly important to study and design 3DOM structure for soot combustion.

### Possible Reaction Mechanism for Soot Combustion on K-OMS-2/SiO_2_ Catalyst

**Figure 14:**
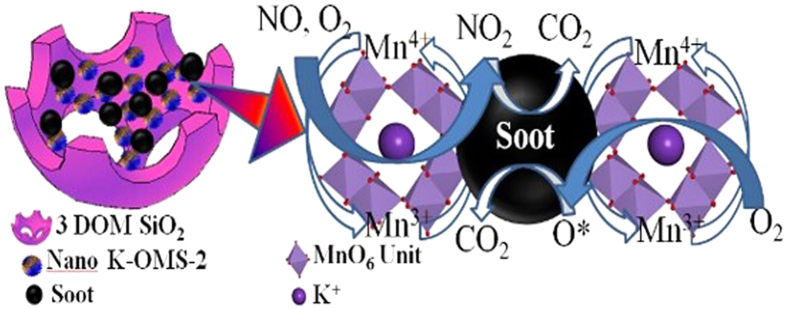
The reaction mechanisms of 3DOM K-OMS-2/SiO_2_ catalyst for soot combustion.

Based on all the results and analyses above, the potential mechanism schemes for soot combustion under presence/absence of NO are proposed and the possible mechanisms are shown in Fig. 14. When the NO is absent in the reaction system, soot oxidation occurred on the interface of K-OMS-2 nanoparticles and soot by the active oxygen species, which can be continuously supplemented by gaseous O_2_ through the oxygen vacancies. Firstly, the gaseous O_2_ is adsorbed on the active sites (i.e. oxygen vacancies on the surface of K-OMS-2), and then the adsorbed O_2_ is decomposed by redox reaction of Mn^3+^/Mn^4+^ and formed active oxygen species(AOSs). Due to containing of K^+^ in the tunnels of K-OMS-2, the electron clouds of K^+^ may enhance the redox performance of Mn^3+^/Mn^4+^ and lead to high production of AOSs. Thirdly, the AOSs are released from the surface of K-OMS-2 and migrated to the surface of soot. At last, the AOSs would be reacted with soot^47^. In this section, long distance between soot and K-OMS-2 would result in inactivation of AOSs. However, because of macropores effect of 3DOM structure, most of AOSs are fully exploited due to good contact between soot and K-OMS-2. As mentioned in the previous discussion, the reaction path of soot combustion has changed when NO is presented. The reaction mechanisms for the soot-NO-O_2_ system can be proposed or summarized as follows. (1) The NO is oxidized to NO_2_ by AOSs (released from the surface of K-OMS-2) in the gaseous atmosphere, and then strong oxidizing NO_2_ migrated to the surface of soot and oxidized the soot. This way may be not played a major role in the system of soot-NO-O_2_ due to inactivation of AOSs in the diffusion process; (2) Adsorption of gas phase O_2_ and NO on the surface of catalysts. As revealed by XPS in the Fig. 9, due to changing valence state of Mn species (Mn^3+^/Mn^4+^) in the K-OMS-2, the oxygen vacancies are formed on the surface of catalysts, and then the active oxygen can be easily generated on the vacancies sites and formed chemisorbed oxygen^48^. Correspondingly, the NO was also interacted with surface active oxygen and formed bidentate/monodentate nitrates on the Mn^n+^ sites. Those bidentate/monodentate nitrates species were desorbed by formation of NO_2_ from K-OMS-2, which acts as oxidant species for soot combustion via a spillover mechanism when temperature is increased. In this section, the doping of K^+^ has significant influence on oxygen vacancies and NO adsorption. The intercalated K^+^ leads to the mixed/averaged valence state of Mn^4+^ and Mn^3+^ are randomly distributed and easily transformed between Mn^4+^ and Mn^3+^. The interaction between K^+^ and Mn^n+^ contributes to forming AOSs and enhance catalytic activity^36^. (3) The special channel structure of K-OMS-2, which is larger than 0.46 nm, allows a lot of gas molecules to insert the channels. In this work, the dynamic diameters of NO and O_2_ are 0.317 and 0.346 nm, respectively. Thus, the channel size of K-OMS-2 is suitable for adsorption of NO and O_2_. In the channel of K-OMS-2, O_2_ can be activated by the redox reaction of Mn^3+^/Mn^4+^ and forms AOSs, and then the NO will be oxidized by AOSs to form NO_2_. After that, the NO_2_ is spread out from the tunnel structure and oxidizes the soot. In this section, a mass of gaseous NO molecules are spread into the tunnel structure owing to the adsorption of K^+^. Therefore, a great number of gaseous NO molecules can be transformed into NO_2_ to enhance the catalytic activity. More importantly, the K^+^ ions are trapped in the 2 × 2 channels of K-OMS-2^49^. This structure guaranteed the stabilization of K^+^ in the soot combustion. In the current work, as discussed above, the doping K^+^ in the manganese oxides plays an essential role in the high catalytic activity for soot combustion.

## Conclusions

A series of novel ordered micro/macro porous K-OMS-2/SiO_2_ oxide catalysts, which contain 3DOM structure in the SiO_2_ support and microporous structure in the nano-K-OMS-2, were firstly designed and successfully prepared by a very simple method. The average diameter of 3DOM SiO_2_ is about 310 nm and microporous K-OMS-2 is about 0.46 nm. More interestingly, the microporous K-OMS-2 oxide nanoparticles with 20–25 nm are well dispersed and supported on the inner wall of the uniform macropores of SiO_2_.

The as-prepared catalysts were firstly used in soot combustion reaction and they show high catalytic activities. Especially, the catalytic activities of as-prepared K-OMS-2/SiO_2_ catalysts are similar to or even higher than those of the expensive noble metal catalysts in the medium and high temperature range. For example, the T_50_ of K-OMS-2/SiO_2_-50, 328 °C, is much lower than those of Pt/Al_2_O_3_ and 3DOM Au/LaFeO_3_, 464 and 356 °C, respectively. Moreover, the catalysts exhibited high catalytic stability for soot combustion. The macroporous effect of 3DOM structure is responsible for increasing the contact efficiency, the microporous effect of 2 × 2 tunnels of K-OMS-2 for adsorption of gas molecules and interaction of K and Mn for the activation of gas molecules, which are favorable for enhancing the catalytic activity for soot combustion. The K^+^ ions are inevitably introduced into the microporous structure of OMS-2 and stabilized in the catalytic reaction. Meanwhile, the K^+^ ions play an important role in templating and stabilizing the tunneled framework of OMS-2. The characterization results also prove that the catalytic activity of ordered hierarchical micro/macro porous K-OMS-2/SiO_2_ catalyst is similar to that of fresh catalyst after five-cycle reaction.

Via the strategy of changing of K-OMS-2 loading and comparison of different active components, we successfully prepared a low cost, environmentally friendly, and highly active catalyst for soot combustion. The as-prepared catalysts are comparable to the expensive noble metal catalysts. This work should aid the rational design and facile preparation of highly efficient oxidation catalysts through the incorporation of other different cations into the tunnel of K-OMS-2. More importantly, the as-prepared micro/macro porous K-OMS-2/SiO_2_ catalysts are promising for practical applications in the catalytic oxidation of diesel soot particles due to easy synthesis, low cost, high activity and stability.

## Methods

### Synthesis of Highly Well-defined PMMA Microspheres

The PMMA microspheres were synthesized by a modified emulsifier-free emulsion polymerization method^14^. The detailed synthesis steps are listed in the Supporting Information. (SEM images of PMMA microspheres and PMMA colloidal crystal templates are shown in Fig. S1).

### Synthesis of 3DOM SiO_2_

3DOM SiO_2_ was synthesized by colloidal crystal template (CCT) method with PMMA arrays as template and using tetraethyl orthosilicate (TEOS) as precursors^50^. In a typical procedure, 4.16 g TEOS was dissolved into the mixture of 2.5 mL water, 5 mL alcohol and 2.5 mL HCl aqueous solution (2 mol/L). After that, the hydrolyzation was proceeded in a water bath at 35 °C for 4 h. Then, 3 g PMMA arrays were impregnated into the above solution for 2 h. After complete impregnation, the PMMA arrays with the precursor solution were separated by vacuum filter and dried at 30 °C for 24 h. The dried samples were calcined to remove the CCT in a tube furnace with an air flow (80 ml min^−1^). The temperature-rising rate was 1 °C min^−1^ from room temperature to 600 °C, and the temperature of calcination at 600 °C was kept for 4 h, and then 3DOM SiO_2_ supports were obtained.

### Synthesis of 3DOM SiO_2_-supported Microporous K-OMS-2 NPs

3DOM K-OMS-2/SiO_2_ catalysts were synthesized by incipient wetness impregnation method. In a typical procedure, a certain amount of KNO_3_ and Mn(NO_3_)_2_ aqueous solution(50 wt%) were dissolved into deionized water, and then the above mixed aqueous solution was added into 3DOM SiO_2_. In this step, the volume of KNO_3_ and Mn(NO_3_)_2_ mixed aqueous solution should be equal to the pore volume of 3DOM SiO_2_. After that, the impregnated sample was dealt with ultrasonic for 10 min and dried at 80 °C for 24 h. Then, the sample was calcined at 550 °C for 4 h in tube furnace and 3DOM K-OMS-2/SiO_2_ catalysts were obtained. In order to obtain different K-OMS-2 loading amounts for K-OMS-2/SiO_2_ catalysts, the weight ratio of K-OMS-2 to SiO_2_ was changed. The as-prepared catalysts were defined as K-OMS-2/SiO_2_-10, K-OMS-2/SiO_2_-20 and K-OMS-2/SiO_2_-30 for corresponding mass ratios of KNO_3_ to SiO_2_ were 5%, 10% and 15%, respectively. The dosages of raw materials are listed in the Table S1.

### Synthesis of 3DOM MnO_x_/SiO_2_, KNO_3_/SiO_2_ and Powder K-OMS-2/silica gel

The detailed synthesis steps are described in the Supporting information.

### Physical and Chemical Characterization

The characterization methods of X-ray diffraction (XRD), scanning electron microscopy (SEM), transmission electron microscopy (TEM), thermogravimetric analysis and differential scanning caborimetry (TG-DSC), Raman spectroscopy, H_2_ temperature-programmed reduction (H_2_-TPR) and X-ray photoelectron spectroscopy (XPS) are described in the Supporting information.

### Activity Measurements

The catalytic performances of all the catalysts were evaluated with a temperature-programmed oxidation reaction (TPO) on a fixed-bed tubular quartz reactor (Φ = 8 mm), and each TPO run from 150 to 650 °C at a 2 °C min^−1^ rate. The model soot was Printex-U particulates (diameter 25 nm, purchased from Degussa). Elemental analysis of Printex-U particulates showed its carbonaceous nature with 92.0% C, 0.7% H, 3.5% O, 0.1% N, 0.2% S and 3.5% others^14^. The catalyst (100 mg) and soot (10 mg) were mixed at a weight ratio of 10:1 with a spatula in order to reproduce the loose contact mode. Reactant gases (50 mL min^−1^) contain 10% O_2_ and 0.2% NO balanced with Ar. The outlet gas compositions were analyzed with an on-line gas chromatograph (GC, Sp-3420, Beijing) by using FID detectors. Before entering the FID detector, CO and CO_2_ were fully converted to CH_4_ by a convertor with Ni catalyst at 380 °C. The catalytic activity was evaluated by the values of T_10_, T_50_ and T_90_, which were defined as the temperatures at 10%, 50% and 90% of soot conversion, respectively. The selectivity to CO_2_ formation (S_CO2_) was defined as that the CO_2_ outlet concentration (C_CO2_) divided by the sum of the CO_2_ and CO outlet concentration, i.e., S_CO2_ = C_CO2_/(C_CO_ + C_CO2_). S^m^_CO2_ was denoted as S_CO2_ at the maximum temperature corresponding to the soot-burnt rate was the highest. In all TPO experiments, the reaction was not finished until the soot was completely burnt off.

## Additional Information

**How to cite this article:** Yu, X. *et al*. Ordered micro/macro porous K-OMS-2/SiO_2_ nanocatalysts: Facile synthesis, low cost and high catalytic activity for diesel soot combustion. *Sci. Rep.* **7**, 43894; doi: 10.1038/srep43894 (2017).

**Publisher's note:** Springer Nature remains neutral with regard to jurisdictional claims in published maps and institutional affiliations.

## Supplementary Material

Supplementary Information

## Figures and Tables

**Table 1:** Surface compositions and oxidation states of K, Mn, O and Si species calculated from XPS analyses.

Catalysts	Mn 2p R%		R%			R%	
	Mn^3+^	Mn^4+^	O-I	O-II	O-III	Si^4+^	Si-OH
K-OMS-2/SiO_2_-20	63.1	36.9	7.3	90.5	2.2	96.9	3.1
K-OMS-2/SiO_2_-50	65.9	34.1	14.9	82.7	2.4	97.9	2.1
K-OMS-2/SiO_2_-70	68.1	31.9	17.3	80.2	2.5	98.1	1.9
K-OMS-2/silica gel-50	66.3	33.7	11.5	85.8	2.7	95.7	4.3
MnOx/SiO_2_-50	62.3	37.7	10.1	87.7	2.2	97.1	2.9
KNO_3_/SiO_2_-50	—	—	3.7	92.8	3.5	98.4	1.6

R%: the ratio of single component to total component.

**Table 2:** Catalytic performances of as-prepared catalysts for soot combustion.

Catalysts	T_10_/°C	T_50_/°C	T_90_/°C	S_CO2_^m^/%	ΔT_10_	ΔT_50_	ΔT_90_
pure soot	482	564	609	71.6	—	—	—
3DOM SiO_2_	354	503	550	78.1	128	61	59
K-OMS-2/SiO_2_-10	315	380	420	93.5	167	184	189
K-OMS-2/SiO_2_-20	310	367	402	94.3	172	197	207
K-OMS-2/SiO_2_-30	296	346	382	94.7	186	218	227
K-OMS-2/SiO_2_-40	290	337	369	95.1	192	227	240
K-OMS-2/SiO_2_-50	283	328	363	96.7	199	236	246
K-OMS-2/SiO_2_-60	288	333	366	96.3	194	231	243
K-OMS-2/SiO_2_-70	286	330	360	96.5	196	234	249
K-OMS-2/silica gel-50	336	381	423	96.1	146	183	186
MnO_*x*_/SiO_2_-50	301	359	397	96.9	181	205	212
KNO_3_/SiO_2_-50	312	365	389	92.5	170	199	220

∆T_10_: The difference value of T_10_ between pure soot and catalysts, ∆T_50_: The difference value of T_50_ between pure soot and catalysts, ∆T_90_: The difference value of T_50_ between pure soot and catalysts.

**Table 3:** Comparison between reported catalysts in referees and as-prepared catalyst for soot combustion under loose contact conditions.

Catalysts	Reaction conditions		m_1_/g^a^	m_2_/g^b^	T_10_/°C	T_50_/°C	T_90_/°C	S_CO2_^m^/%	Refs
	NO/ppm	O_2_/%							
MnO_x_	500	5	0.02	0.08	350	430	480	—	51
Ag/MnO_x_	0	50	0.04	0.36	—	498	—	—	52
Ba/MnO_x_-CeO_2_	1000	10	0.01	0.1	362	393	—	99	53
La_1.6_Rb_0.4_CuO_4-λ_	2000	5	0.01	0.1	429	505		96.3	54
KCu_2_/Al_2_O_3_	600	5	0.125	0375	—	480	—	—	55
Pt/Al_2_O_3_	1000	10	0.01	0.1	—	464	—	>98	56
3DOM LaCo_0.5_ Fe_0.5_O_3_	2000	5	0.01	0.1	256	397	436	99.7	57
3DOM Ce_0.8_Zr_0.2_O_2_	2000	5	0.01	0.1	348	396	424	—	58
3DOM Au_0.08_/LaFeO_3_	2000	5	0.01	0.1	229	359	—	99.7	14
3DOM Au_0.04_/Ce_0.8_Zr_0.2_O_2_	2000	5	0.01	0.1	218	356	404	99.7	59
3DOM Pt_0.08_/Ce_0.8_Zr_0.2_O_2_	2000	5	0.01	0.1	248	326	369	98.1	60
3DOM Au_2_@Pt_2_/Ce_0.8_Zr_0.2_O_2_	2000	5	0.01	0.1	214	325	368	99.9	61
3DOM K-OMS-2/SiO_2_-50	2000	10	0.01	0.1	283	328	363	96.7	This work

^a^mass of soot.

^b^mass of catalyst.
