# Supplementary material for: Ordered micro/macro porous K-OMS-2/SiO2 nanocatalysts: Facile synthesis, low cost and high catalytic activity for diesel soot combustion
Source: Sci Rep. 2017 Apr 26;7:43894. doi: 10.1038/srep43894 (PMC5405416; doi:10.1038/srep43894)
Supplement: Supplementary Information [file srep43894-s1.doc]

Supporting information for

Ordered micro/macro porous K-OMS-2/SiO_2_ nanocatalysts: Facile synthesis, low cost and high catalytic activity for diesel soot combustion

Xuehua Yu^1^, Zhen Zhao^1,2*^, Yuechang Wei^2^, Jian Liu^2^

^1^ Institute of Catalysis for Energy and Environment, College of Chemistry and Chemical Engineering, Shenyang Normal University, Shenyang, Liaoning, 110034, China

^2^ State Key Laboratory of Heavy Oil Processing, China University of Petroleum, Beijing,

18# Fuxue Road, Chang Ping, Beijing, 102249, China

Synthesis of Highly Ordered PMMA Colloidal Crystal Microspheres

The PMMA colloidal crystal microspheres were synthesized by a modiﬁed emulsiﬁer-free emulsion polymerization approach. In a typical procedure, 240 mL of deionized water was added into a four orifices reactor, which was heated by a hot water bath at 80 ^o^C and blown Ar as protection gas. Then, a water-cooling condenser and mechanical stirrer were ﬁxed to the reactor. After that, 120 mL methyl methacrylate monomer was poured into the reactor through the four opening which was otherwise closed with a stopper. After stirring for 20 min, a solution of potassium persulfate initiator (0.60 g dissolved in 40 mL of deionized water) preheated to 80 ^o^C was added into the reactor. With Ar protection and mechanical stirring, the reaction was allowed to react at 80 °C for 2 h, and then the emulsion was filtered by microfiltration membrane. In order to get PMMA colloidal crystal microspheres, the filtrate was assembled by centrifugation at 3000 rpm for 10 h, the clear liquid was decanted and the solid block was dried at 30 ^o^C for 24 h. Last, the highly ordered PMMA arrays were obtained. The PMMA colloidal crystal microspheres are ca. 440 nm in average diameter (Figure S1).


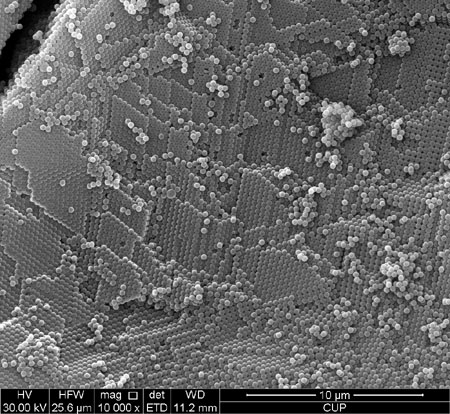

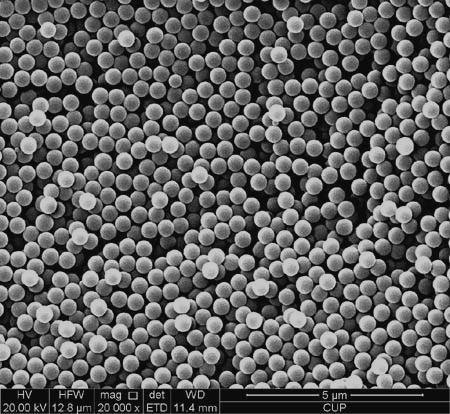


a

b

Figure S1 SEM images of PMMA microspheres (a) and PMMA colloidal crystal templates(b)

(Insert is histograms of PMMA microspheres)

Synthesis of 3DOM MnO_x_/SiO_2_, KNO_3_/SiO_2_ and Powdered K-OMS-2/silica gel

In order to deeply investigate catalytic activity of K-OMS-2 and macropore effect for soot combustion, 3DOM MnO*_x_*/SiO_2_, KNO_3_/SiO_2_ and powdered K-OMS-2/silica gel were also synthesized by incipient wetness impregnation method. The molar amount of Mn(NO_3_)_2_ in MnO*_x_*/SiO_2_-50 equals to total molar amount of Mn(NO_3_)_2_ and KNO_3_ in the K-OMS-2/SiO_2_-50 (the same molar amount of KNO_3_ in the KNO_3_/SiO_2_-50). The powder K-OMS-2/silica gel-50 is adopted silica gel as support. The other detail synthesis steps are similar to those of 3DOM K-OMS-2/SiO_2_ catalyst.

Table S1 Expression ways of stoichiometric ratio for as-prepared catalysts

| Catalysts | ^a^ KNO_3_/SiO_2_ | K:Mn | KNO_3_/g | ^b^Mn(NO_3_)_2_/g | 3DOM SiO_2_/g |
| --- | --- | --- | --- | --- | --- |
| K-OMS-2/SiO_2_-10 | 5% | 1:2 | 0.025 | 0.167 | 0.500 |
| K-OMS-2/SiO_2_-20 | 10% | 1:2 | 0.050 | 0.335 | 0.500 |
| K-OMS-2/SiO_2_-30 | 15% | 1:2 | 0.075 | 0.501 | 0.500 |
| K-OMS-2/SiO_2_-40 | 20% | 1:2 | 0.100 | 0.671 | 0.500 |
| K-OMS-2/SiO_2_-50 | 25% | 1:2 | 0.125 | 0.835 | 0.500 |
| K-OMS-2/SiO_2_-60 | 30% | 1:2 | 0.150 | 1.002 | 0.500 |
| K-OMS-2/SiO_2_-70 | 35% | 1:2 | 0.175 | 1.170 | 0.500 |
| MnO*_x_*/SiO_2_-50 | 0% | 0 | 0 | 1.252 | 0.500 |
| KNO_3_/SiO_2_-50 | 75% | -- | 0.375 | 0 | 0.500 |
| K-OMS-2/Silica gel-50 | 25% | 1:2 | 0.125 | 0.835 | ^c^0.500 |

a: Weight ratio of KNO_3_ to SiO_2_

b: 50 wt% Mn(NO_3_)_2_ aqueous solution

c: Silica gel with shape of particles

Physical and Chemical Characterization

XRD patterns were measured on a powder X-ray diffractometer (Bruker D8 Advance) using CuKa (k=0.15406 nm) radiation with a Nickel ﬁlter operating with voltage and current of 40 kV and 40 mA in the 2θ range of 10-90^o^ at a scanning step of 0.02. The patterns were compared with JCPDS reference data for phase identiﬁcation. The surface morphology of the catalyst was observed by ﬁeld emission scanning electron microscopy (FESEM) on a Quanta 200F instruments using accelerating voltages of 5 kV. The samples for SEM measurements were dusted on conducting resin and coated with 10 nm Au prior to measurement. The TEM and HRTEM images were obtained using a JEOL JEM-2100 transmission electron microscope. A typical TEM sample was prepared by adding several droplets of a nanoparticles/ethanol mixture onto a carbon-coated copper grid. Raman spectra were obtained in a backscattering conﬁguration on a Renishaw 1000 confocal laser Raman spectrometer with 8 mW Ar^+^ laser (532 nm) and a ×40 long-focus lens and the time of acquisition was 10s. The thermal stability was assessed by thermogravimetric analysis and differential scanning caborimetry (TGA-DSC), which were performed on a Netzsch STA409PC thermal gravimetric analyzer in dry air with a heating rate of 10 ^o^C/min.

H_2_-TPR measurements were performed using a Quantachrome Autosorb-iQ, USA. A sample of 100 mg was loaded into a U-shaped quartz reactor and pre-treated in Ar at 573 K for 1 h. After cooling to room temperature, the flow gas was switched to 10-vol% H_2_/Ar, and the catalyst was heated to 1173 K at a rate of 10 K min^-1^. The consumption of hydrogen was recorded by thermal conductivity detector (TCD). Calibration of the instrument was carried out with CuO of known amount. X-ray photoelectron spectra (XPS) were recorded on a Perkin-Elmer PHI-1600 ESCA spectrometer using Mg Kα X-ray source. The catalysts were treated at 150 ^o^C for 48 h in the vacuum drying oven in order to exclude the hydroxyl and carbonate oxygen on the surface of catalysts. The binding energies were calibrated using C1s peak of contaminant carbon (BE = 284.6 eV) as an internal standard.

**Table S2.** Binding energies of K, Mn, O and Si in as-prepared catalysts obtained from XPS

| Catalysts | BE (eV) | | | | | | | | | | |
| --- | --- | --- | --- | --- | --- | --- | --- | --- | --- | --- | --- |
|  | K 2p | | Mn 2p_3/2_ | | Mn 2p_1/2_ | | O1s | | | Si 2p | |
|  | K 2p_3/2_ | K 2p_1/2_ | Mn^3+^ | Mn^4+^ | Mn^3+^ | Mn^4+^ | O-I | O-II | O-III | Si^4+^ | Si-OH |
| K-OMS-2/SiO_2_-20 | 292.6 | 295.4 | 641.9 | 644.8 | 653.5 | 656.0 | 529.6 | 532.7 | 534.9 | 103.4 | 105.6 |
| K-OMS-2/SiO_2_-50 | 292.4 | 295.3 | 641.5 | 644.5 | 653.1 | 655.8 | 529.4 | 532.2 | 534.7 | 102.8 | 105.3 |
| K-OMS-2/SiO_2_-70 | 292.6 | 295.4 | 641.4 | 644.4 | 652.9 | 655.7 | 529.4 | 532.3 | 534.7 | 103.1 | 105.4 |
| K-OMS-2/silica gel-50 | 292.5 | 295.4 | 641.6 | 644.6 | 653.2 | 655.7 | 529.8 | 532.4 | 534.6 | 103.2 | 105.4 |
| MnOx/SiO_2_-50 | -- | -- | 641.5 | 644.2 | 653.4 | 656.0 | 529.2 | 532.6 | 534.8 | 103.4 | 105.5 |
| KNO_3_/SiO_2_-50 | 292.7 | 295.6 | -- | -- | -- | -- | 529.4 | 532.3 | 534.7 | 103.1 | 105.3 |


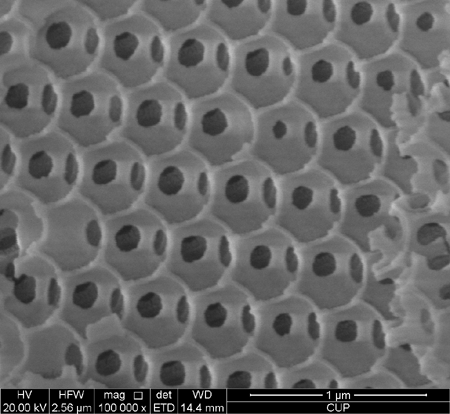

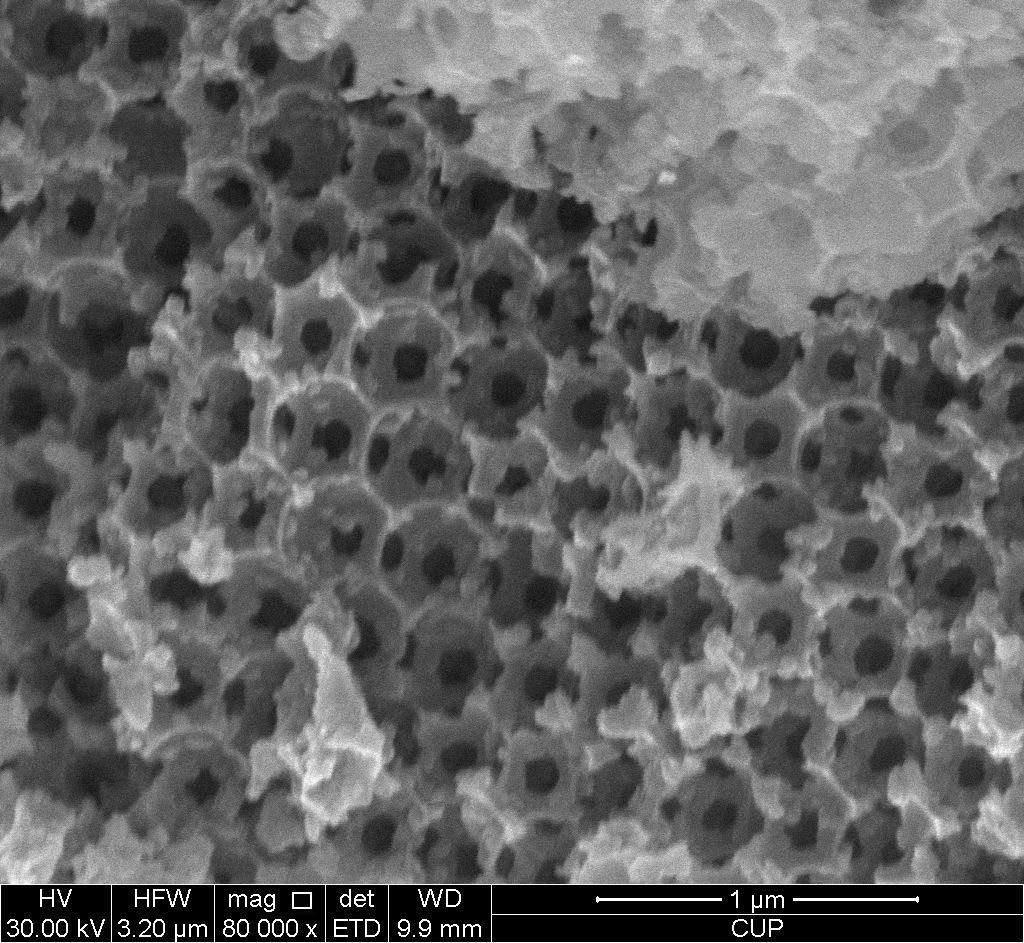

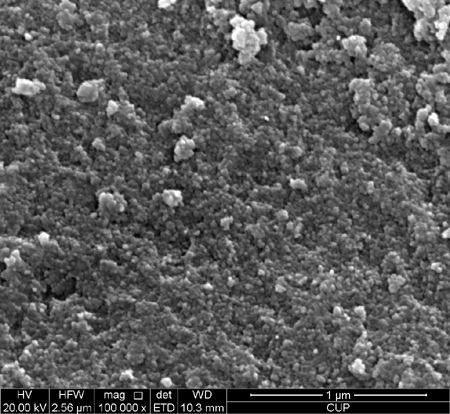


a

b

c

Figure S2 SEM images of as-prepared catalysts with varied supports and active components: K-OMS-2/silica gel-50 (a), MnO_x_/SiO_2_-50 (b), KNO_3_/SiO_2_-50(c)


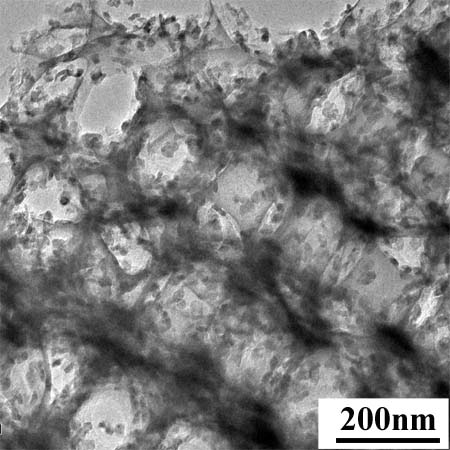

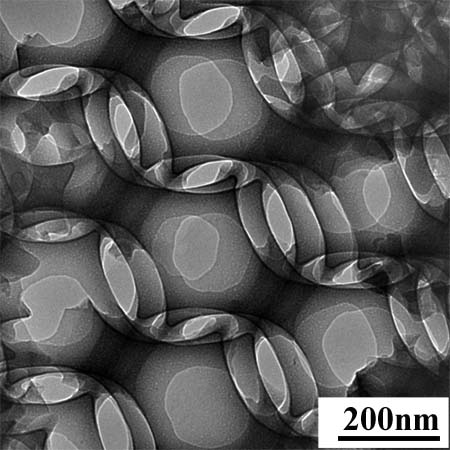

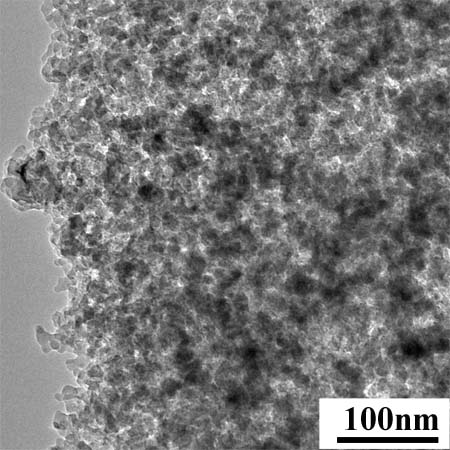


a

b

c

Figure S3 TEM images of as-prepared catalysts with varied supports and active components: K-OMS-2/silica gel-50 (a), MnO_x_/SiO_2_-50 (b), KNO_3_/SiO_2_-50(c)


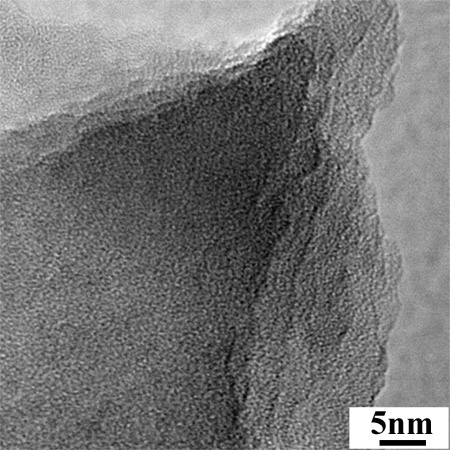

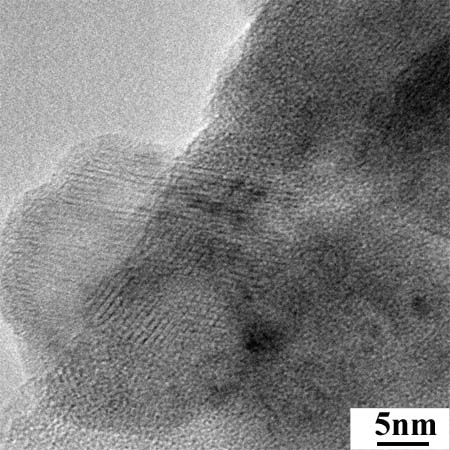

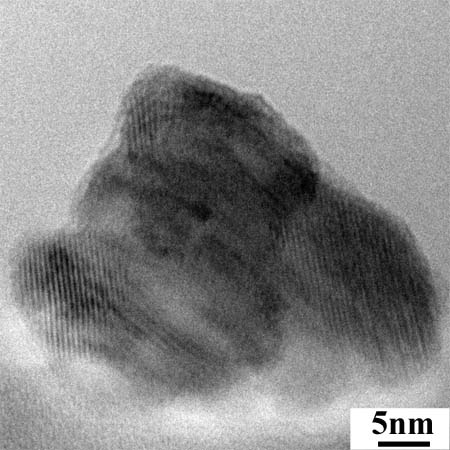

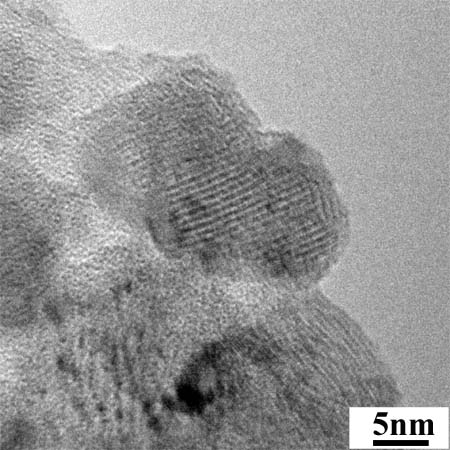

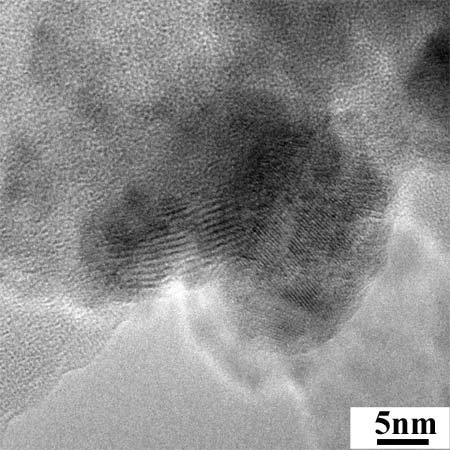

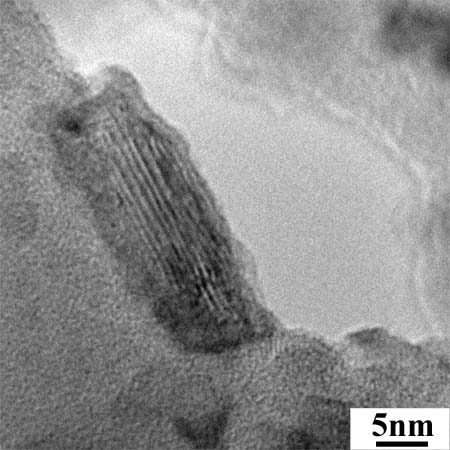

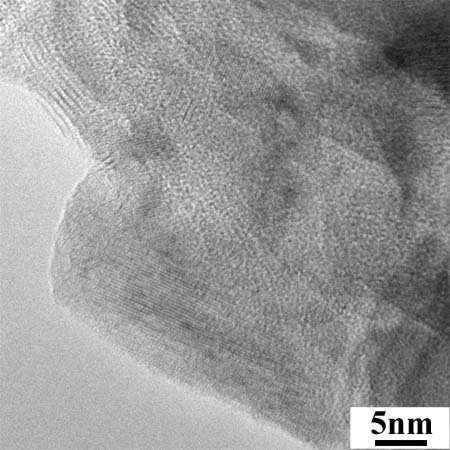


e

f

c

d

b

a

g

Figure S4 HRTEM images of 3DOM K-OMS-2/SiO_2_ catalysts

(K-OMS-2 loadings: SiO_2_ (a), K-OMS-2/SiO_2_-10 (b), K-OMS-2/SiO_2_-20 (c), K-OMS-2/SiO_2_-30 (d), K-OMS-2/SiO_2_-40 (e), K-OMS-2/SiO_2_-60 (f), K-OMS-2/SiO_2_-70 (g)

b

a

c

d

e

f

g

h

Figure S5 Histograms of K-OMS-2 nanoparticles in K-OMS-2/SiO_2_ catalysts and MnOx/SiO_2_. K-OMS-2 loadings: K-OMS-2/SiO_2_-10 (a), K-OMS-2/SiO_2_-20 (b), K-OMS-2/SiO_2_-30 (c), K-OMS-2/SiO_2_-40 (d), K-OMS-2/SiO_2_-50 (e), K-OMS-2/SiO_2_-60 (f), K-OMS-2/SiO_2_-70 (g), MnO_x_/SiO_2_-50 (h)
